# Transcriptional landscape of the embryonic chicken Müllerian duct

**DOI:** 10.1186/s12864-020-07106-8

**Published:** 2020-10-02

**Authors:** Zahida Yesmin Roly, Rasoul Godini, Martin A. Estermann, Andrew T. Major, Roger Pocock, Craig A. Smith

**Affiliations:** Department of Anatomy and Developmental Biology, Monash Biomedicine Discovery Institute, Monash University, Wellington Road, Clayton, VIC 3800 Australia

**Keywords:** Müllerian duct, Chicken embryo, RNA-seq, *FOXE1*, *OSR1*, Sex determination

## Abstract

**Background:**

Müllerian ducts are paired embryonic tubes that give rise to the female reproductive tract in vertebrates. Many disorders of female reproduction can be attributed to anomalies of Müllerian duct development. However, the molecular genetics of Müllerian duct formation is poorly understood and most disorders of duct development have unknown etiology. In this study, we describe for the first time the transcriptional landscape of the embryonic Müllerian duct, using the chicken embryo as a model system. RNA sequencing was conducted at 1 day intervals during duct formation to identify developmentally-regulated genes, validated by in situ hybridization.

**Results:**

This analysis detected hundreds of genes specifically up-regulated during duct morphogenesis. Gene ontology and pathway analysis revealed enrichment for developmental pathways associated with cell adhesion, cell migration and proliferation, ERK and WNT signaling, and, interestingly, axonal guidance. The latter included factors linked to neuronal cell migration or axonal outgrowth, such as Ephrin B2, netrin receptor, SLIT1 and class A semaphorins. A number of transcriptional modules were identified that centred around key hub genes specifying matrix-associated signaling factors; *SPOCK1, HTRA3* and *ADGRD1*. Several novel regulators of the WNT and TFG-β signaling pathway were identified in Müllerian ducts, including *APCDD1* and *DKK1*, *BMP3* and *TGFBI*. A number of novel transcription factors were also identified, including *OSR1, FOXE1, PRICKLE1, TSHZ3* and *SMARCA2*. In addition, over 100 long non-coding RNAs (lncRNAs) were expressed during duct formation.

**Conclusions:**

This study provides a rich resource of new candidate genes for Müllerian duct development and its disorders. It also sheds light on the molecular pathways engaged during tubulogenesis, a fundamental process in embryonic development.

## Background

Two pairs of ducts form during embryonic development in vertebrates; the Wolffian and Müllerian ducts. In male embryos, Wolffian ducts develop into the male reproductive tract, under the influence of androgens produced by the testis [1]. Another testis-derived factor in males, Anti-Müllerian Hormone (AMH) induces regression of the Müllerian ducts [2]. In female embryos, the converse applies. The absence of testosterone leads to regression of the Wolffian ducts, and the absence of fetal AMH expression allows the Müllerian ducts to form the female reproductive tract (Fallopian tubes, uterus and upper vagina) [3]. In humans, disorders of Müllerian duct development during embryogenesis lead to anomalies of the reproductive tract. Disorders can include abnormal retention of the ducts in males (Persistent Müllerian Duct Syndrome) [4, 5] and developmental defects of duct development in females [6]. Mayer-Rokitansky-Küster-Hauser (MRKH) syndrome (OMIM 277000) is characterised by dysplasia of the uterus and upper vagina [7–10]. MRKH is estimated to occur in up to 1 in 4500 women and the condition can be isolated (type I) or associated with other (non-reproductive) congenital malformations (type II) [9, 11]. While evidence from familial studies indicates that MRKH has a likely genetic basis, its molecular aetiology is poorly understood. Some chromosomal deletions have been associated with MRKH that include loss-of-function mutations in genes such as *LIM1 (LIM Homeobox 1), WNT4* and *WNT9B*, all of which are critical for embryonic Müllerian duct formation [12–16]. Another Müllerian-linked anomaly, Hand-Foot-Genital (HFG) is associated in some cases with *HOXA13* lesions (duplications or polyalanine expansions) [17, 18]. However, most cases of Müllerian disorders such as MRKH and HFG cannot be explained by mutations in known genes. Therefore, there is a need for greater understanding of the developmental pathways leading to normal Müllerian duct formation and its disorders.

Animal models have proven very useful in improving our understanding of normal and abnormal Müllerian duct development. In mouse and chicken embryos, the Müllerian duct forms from coelomic epithelium in close association with the Wolffian duct on the surface of the embryonic (mesonephric) kidney. In both species, three conserved phases of Müllerian duct development are recognised: specification, invagination and elongation [19–22]. Specification occurs among a restricted population of coelomic epithelial cells at the anterior pole of the mesonephros. These cells invaginate to from a mesoepithelial tube that migrates caudally via cell proliferation. Some cells from the coelomic epithelium also undergo an EMT (epithelial to mesenchyme transition), contributing mesenchyme around the elongating duct [23, 24]. These developmental processes are regulated by the coordinated action of several transcription factors and signaling molecules, although their exact roles are not yet fully understood. Homeodomain transcription factors and members of the WNT family of secreted growth factors play essential roles in duct specification, invagination or elongation [22, 25–29]. The homeobox genes, *Lim1* and *Pax2*, are expressed during duct specification and invagination and deletion of these genes prevents Müllerian duct formation [20, 25, 27, 28]. Wnt4 is expressed in the mesenchyme of the duct and is required for proper duct formation in rodent models [22]. Downstream of Wnt4, other Wnt family members (Wnt7a, Wnt5a, Wnt9b) are important for tubulogenesis and caudal extension of the duct [24, 28, 30–35]. In addition to the pervasive role of Wnt signaling, retinoic acid (RA) signaling is also required for Müllerian duct development and differentiation [36]. Mice lacking both RA receptors α and β2 lack Müllerian ducts [37, 38]. However, the exact role of RA signaling during duct formation is unclear.

To gain greater insight into the genes and developmental pathways regulating Müllerian duct formation, we conducted RNA-sequencing (RNA-seq), using the chicken embryo as a model system. Müllerian duct development is conserved between chicken and mammals, involving the same specification, invagination and elongation phases [21, 23, 39]. Genes known to be involved in early duct formation in mouse, such as *Lim1* and *Pax2* and *Wnt4*, are also implicated in the chicken [21, 40]. This is the first report describing the transcriptional landscape of the Müllerian duct in any species. Transcriptome analysis reveals the molecular genetic modules activated during duct formation and elongation, with enriched “hub genes” involved in matrix-associated signaling. We find enrichment of developmental pathways associated with cell adhesion, cell migration and proliferation, ERK and WNT signaling, and axonal guidance. We report several novel candidate regulators of Mullerian duct formation, including the transcription factors *OSR1 (*Odd-Skipped Related Transcription Factor 1)*, FOXE1 (*Forkhead Box E1)*, PRICKLE (*Prickle Planar Cell Polarity Protein 1)*, TSHZ3 (*Tea-shirt Zinc Finger Homeobox 3) and *SMARCA2 (*SWI/SNF Related, Matrix Associated, Actin Dependent Regulator Of Chromatin, Subfamily A, Member 2). The datasets also reveal a rich network of long non-coding RNAs expressed in the developing Mullerian duct. These data provide new information on the genetic regulation of tubulogenesis, and development of the Müllerian duct in particular. Based on expression profiling, this study identifies new candidate genes for human Mullerian duct disorders.

## Results

### Overview

**Fig. 1:**
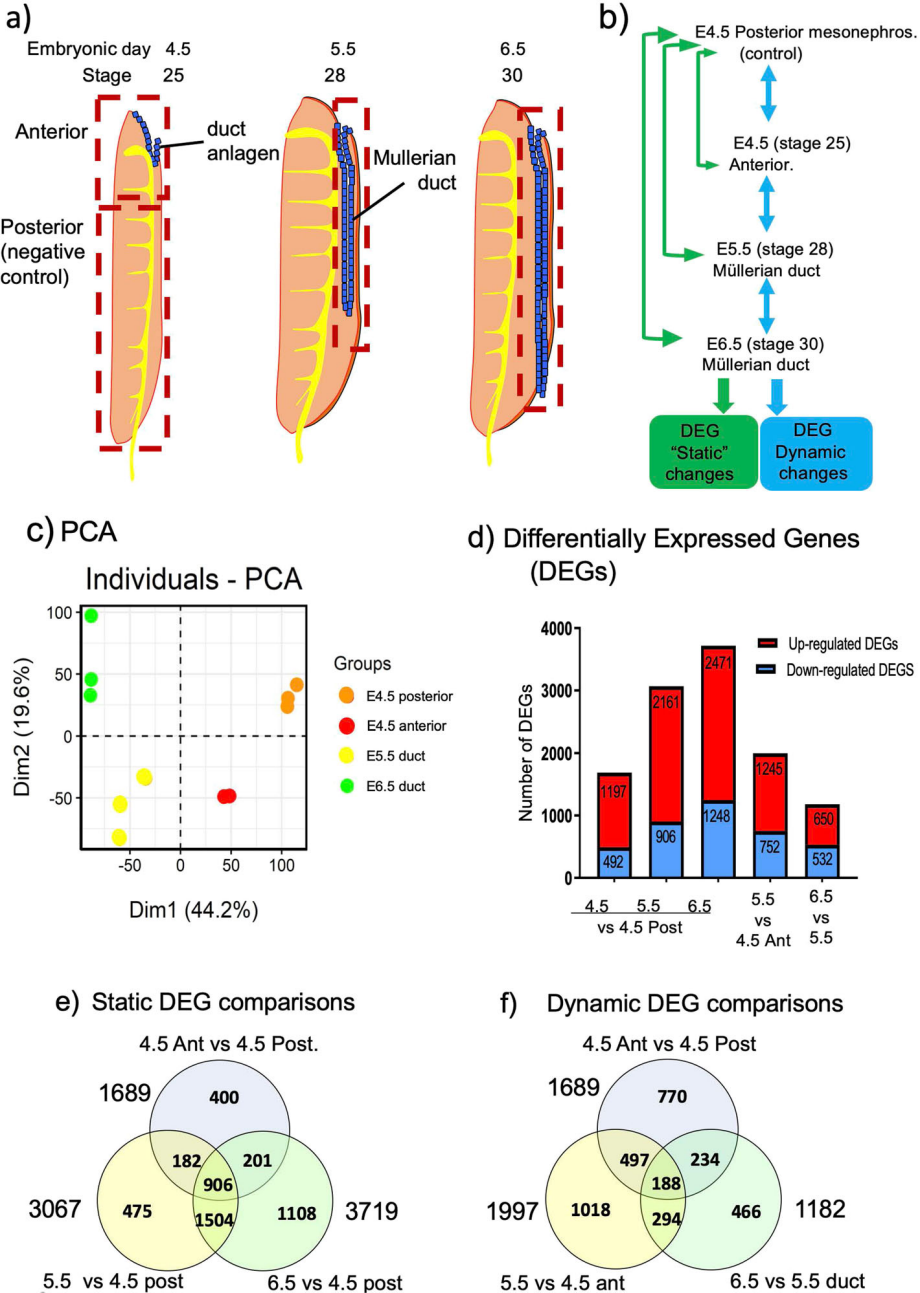
Schematic figure of chicken embryo Müllerian duct tissue sampling and pipeline of the bioinformatical analysis. **a** Samples were taken on 3 consecutive days, E4.5, E5.5 and E6.5. Posterior mesonephros at day 4.5 was used as control (red square). The 4.5 anterior sample comprised both duct and mesonephric kidney tissue. Pure Müllerian duct tissue was collected only at E5.5 and E6.5. **b** Pipeline of RNA-seq bioinformatical analysis. For static comparisons, differentially expressed genes (DEGs) were identifed by comparing each stage to the control tissue (E4.5 posterior mesonephros) (Green arrows). For dynamic comparisons, DEG’s were identified by comparing successive stages of duct development (blue arrows). The datasets were subjected to a number of bioionformatic analyses, including GO terms, PPI, WCGNA and TF developmental clustering. **c** Principle component analysis (PCA) analysis of all samples using all genes. **d** Differentially Expressed Genes (DEG’s) across samples. Up-regluated genes shown in red, down-regulated genes shown in blue. An increasing number of duct genes were up-regulated relative to the control (E4.5 posteroir) as duct development proceeded. **e** Venn diagrams showing shared DEGs based on static comparisons (E4.5 anterior, E5.5 and E6.5 duct comparsed to control tissue, E4.5 posterior mesonehpric kidney.) 906 genes were differentially expressed in all samples relative to the control. **f** Venn diagrams showing shared DEGs based on dynamic comparisons (comparing successive stages of duct development). One hundred eight genes that showed differential expression across all stages of duct development

High-throughput RNA sequencing (RNA-seq) was used to characterise the transcriptional landscape of the embryonic Müllerian duct during development, using the chicken embryo as a model. Duct formation commences in the chicken between embryonic day (E) 4.0–4.5 (Hamburger and Hamilton stage 23–25) [41]. At this stage, Müllerian progenitors are specified in the coelomic epithelium and undergo invagination at the anterior pole of the mesonephric kidney (Fig. 1a) [21]. As it is challenging to separate the Müllerian anlagen from the mesonephros at this early time point, the entire anterior portion of the mesonephros was taken (= 4.5-Ant.). The posterior portion of the mesonephros (lacking duct) served as negative control tissue (= 4.5-Post.) (Fig. 1a). By E5.5 (stage 28), the duct elongates along the surface of the mesonephros and can be separated from it. At E6.5 (stage 30), duct elongation is complete. For both E5.5 and E6.5, developing Müllerian duct alone was dissected away from the mesonephros. RNA was extracted from these four tissues in triplicate and subjected to bulk RNA-sequencing. After constructing libraries, RNA-seq was performed using the Illumina NextSeq500 platform. The library size was approximately 20 million reads per sample. Differential gene expression analysis was carried out using Voom/Limma [42]. Differential expression (DE) was set using the following cut-offs: False Discovery Rate (FDR) ≤ 0.05 and Log_2_FC ≤ 0.585 or ≥ 0.585 (see Methods). One E4.5 Anterior RNA sample did not pass QC and was omitted from the analysis. Differentially expressed genes (DEG) were identified by comparing stages of duct development (E4.5 vs E5.5 vs E65; dynamic comparisons) and between the Mullerian duct and the negative control (E4.5 Posterior mesonephros; static comparisons). (Fig. 1b).

Principle Component Analysis (PCA) of the RNA-seq data showed that tissues at each stage distinctly clustered together (Fig. 1c). Differentially expressed genes (DEGs) for each stage were extracted from the data and visualized in a bar graph to show genes up- or down-regulated across development (Fig. 1d). In E4.5 (stage 25) anterior tissue (E4.5-Ant.), a total of 1689 genes were differentially expressed compared to the E4.5 posterior control (E4.5-Post). Of these, 1197 were up-regulated and 492 were down-regulated. A larger number of genes were differentially expressed as Müllerian duct development progressed. Three thousand sixty-seven genes were differentially expressed between E5.5 duct vs E4.5 posterior (control tissue), and 3719 for E6.5 Müllerian duct vs E4.5 posterior. Figure 1(e) and (f) shows the data on Venn diagrams. Figure 1e shows differentially expressed genes as a “static comparison”, that is, DEGs at each stage compared to the E4.5 Posterior mesonephros (control tissue). Large numbers of genes showed differential expression, including 906 genes that were differentially expressed at all three duct stages (E4.5 Anterior, E5.5 and E6.5) (Fig. 1e). Figure 1f shows “dynamic” DEG comparison, that is, DEGs compared between successive stages of duct development (E4.5 Anterior vs E4.5 Posterior, E5.5. vs E4.5 Anterior, E6.5 vs E5.5). Fewer DEGs were detected in the dynamic comparison compared to the static comparisons, as expected, as the dynamic changes focussed on duct development over time, whereas the static changes compared ducts to a different tissue, the mesonephros. Comparing tissue that contained duct cells (dynamic changes over development for 4.5 anterior, E5.5 and E6.5), 188 genes were differentially expressed at all three stages (Fig. 1f). These transcript comparisons therefore revealed genes specifically associated with Müllerian duct formation.

The RNA-seq data was validated by confirming enriched expression of known factors for Müllerian duct formation [24, 43]. (Supplementary Figure 1). Transcripts of genes previously shown to be essential for Müllerian duct specification and invagination phases, such as *LIM1* and *PAX2* and *BMPs*, were enriched in the datasets. Genes required for duct elongation and/or differentiation, such as *DMRT1*, *WNTs* and *EMX2*, became up-regulated over development. Finally, genes implicated in duct differentiation, such as *HOXA10* and *HOXD10*, were up-regulated at the latest time point examined, E6.5 (stage 30) (Supplementary Figure 1).

### Gene ontology (GO) analysis

**Fig. 2:**
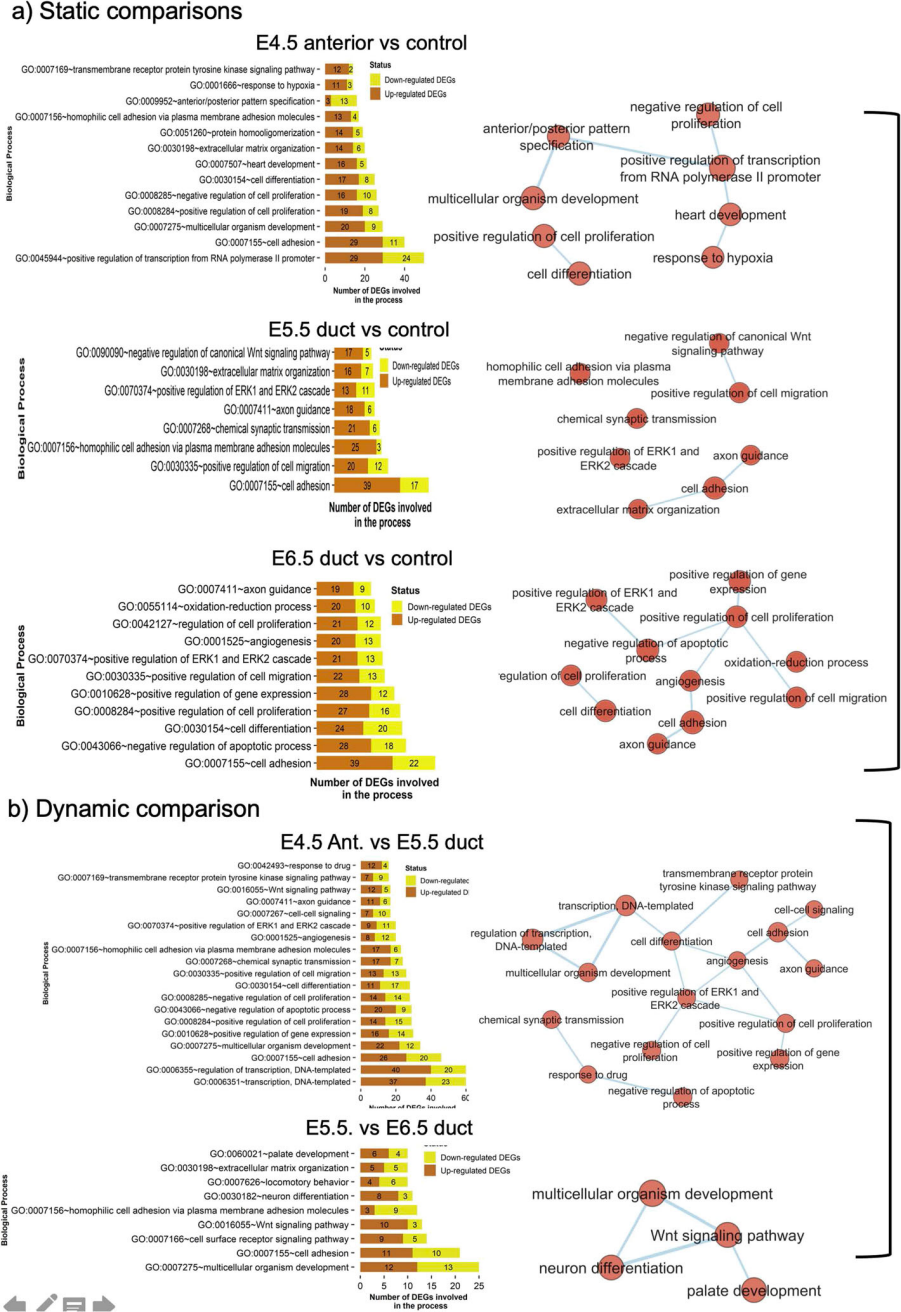
Gene Ontology analysis of DEGs, based on static and dynamic comparisons. **a** Bar plots showing gene ontology-based number of DEG enrichment map analysis to identify enriched pathways in static comparisons (E4.5 Anterior, E5.5 and E6.5 each compared to the control tissue (E4.5 Posterior mesonephros. **b** Bar plots showing gene ontology-based number of DEGs enrichment map analysis to identify enriched pathways in dynamic comparisons (E4.5 Anterior vs E5.5 and E5.5 duct E6.5 each compared to the control tissue (E4.5 Posterior mesonephros

Gene Ontology analysis (GO) reflects how genes are related to a biological process, organ, function or pathway. GO analysis was performed for gene lists from distinct analyses, including DEGs, genes from hierarchical clustering and Weighted Gene Co-expression Network Analysis (WGCNA) (see Methods). Based on static comparisons (E4.5-Ant, E5.5 and E6.5 ducts vs control tissue) gene ontology and pathway analysis revealed enrichment for developmental pathways associated with cell adhesion, cell migration and proliferation, ERK and WNT signaling, and axonal guidance (Fig. 2a and b). In early stages of duct development (4.5 anterior), genes related to transcription and cell differentiation were enriched, while genes linked to cell adhesion and cell proliferation were enriched across all stages of duct formation. Other pathways enriched during duct development included canonical WNT signaling (as expected) and ERK signaling. Transcripts associated with cell migration and axonal guidance were enriched at E5.5 and 6.5, while pathways associated with cell differentiation were enriched especially at E6.5. Transcripts involved in negative regulation of apoptosis were enriched in E6.5 (elongating) ducts (Fig. 2b). In dynamic comparisons (between successive duct stages), mRNAs associated with “multicellular organism development” and transcriptional regulation were enriched, together with WNT signaling and cell adhesion and cell surface receptor signaling (Fig. 2). Interestingly, at E5.5, when the duct could be separated from the mesonephric kidney, processes related to neuronal development or function, such as axon guidance and chemical synaptic transmission were again enriched (Fig. 2a). Finally, WNT pathway signaling was enriched at E6.5 (Fig. 2b).

### Protein-protein interaction (PPI)

Protein-protein interaction (PPI) networks show how proteins encoded by genes physically or functionally interact with each other. Network-based analysis can be used to detect protein groups in the form of sub-networks. PPI networks were constructed for DEGs lists, based on static comparisons (duct tissues compared to E4.5 posterior mesonephros control) (Supplementary Figure 2) and based on dynamic comparisons (ducts over development; E4.5-Post vs E4.5 anterior, E4.5-Ant vs E5.5 duct, E5.5 duct vs E6.5 duct). (Supplementary Figure 3). For static comparisons, GO analysis showed that enriched sub-networks were related to cell adhesion, chemical synaptic transmission, neuron differentiation, and several signaling pathways, including FGF, WNT and G-protein coupled receptor signaling. (Supplementary Figure 2). These enriched GO terms were also present in the dynamic comparisons (Supplementary Figure 3). Comparing networks in E5.5 duct vs E6.5 duct, fewer networks were detected. However, those enriched included the adenylate cyclase inhibiting- and activating- G protein receptor signaling, cell surface antigen processing, muscle cell function. Present in all networks, WNT signaling was consistently up-regulated in all samples, whereas cell adhesion was only up-regulated in comparison with 4.5 posterior tissue (negative control).

### Weighted gene co-expression network analysis for modules

To identify co-expressed genes through Müllerian duct development, we applied WGCNA, which identifies group of genes using network-based analysis and correlation in gene expression. On this basis, a dendrogram clustered the four sample types (Supplementary Figure 4a.) Many clustered modules were initially identified, which could be clustered and merged into groups based on percentage similarity (Supplementary Figure 4b). After applying soft-power 26 and constructing the network by WGCNA, we found 18 modules of co-expressed genes that were correlated or anti-correlated with stages of Müllerian duct development (Supplementary Figure 4c; annotated by different colour codes). Some gene network modules were positively correlated with duct development, for example, the magenta module, enriched over duct development, while others were enriched at E4.5 anterior (dark sea green) or E5.5 (dark-turquoise, ivory, skyblue). Some modules were negatively correlated (for example, dark olive green), revealing networks of genes down-regulated during duct formation. As we aimed to identify genes responsible for Müllerian duct development, we selected 10 modules with positive correlation to duct development for further analysis. Following gene significance vs module membership analysis, we selected eight modules with lowest *P*-value (Supplementary Figure 4c and d). GO analysis was then performed for members of candidate modules to assign biological processes (Supplementary Table 2). The GO results of the modules were in consistent with GO of the DEGs. This weighted gene co-expression network analysis allowed the linkage of gene expression modules to a specific stage. Hence, we found that modules highly correlated with the 4.5 posterior stage were related to DNA replication, intracellular signaling pathways and kidney morphogenesis, which is correlated with the negative control status of this sample (mesonephric kidney). In contrast, the darkseagreen4 and pale-turquoise modules enriched at 4.5 anterior (i.e., kidney plus duct anlagen) (Supplementary Figure 4c), comprised genes related to cell adhesion and actin filament organization. These processes are indeed required for the rearrangement of cells to form the invaginating cells of the early chicken Müllerian duct [21]. At the E5.5 duct stage, enriched modules dark-turquoise and ivory were related to signal transduction, transcription, anterior/posterior pattern specification, and development. These pathways correlated with duct invagination and the early stage of anterior-to posterior caudal elongation. In the more developed E6.5 Mullerian duct, the medium-orchid module was enriched, which was related to translation and protein localisation. The magenta module, also enriched at E6.5, was associated with a variety of biological processes from transcription to signal transduction and development. This module was highly anti-correlated at the 4.5 posterior stage (the negative control), which comprised kidney tissue, suggesting members of this module are highly specific for Mullerian duct development.

**Fig. 3:**
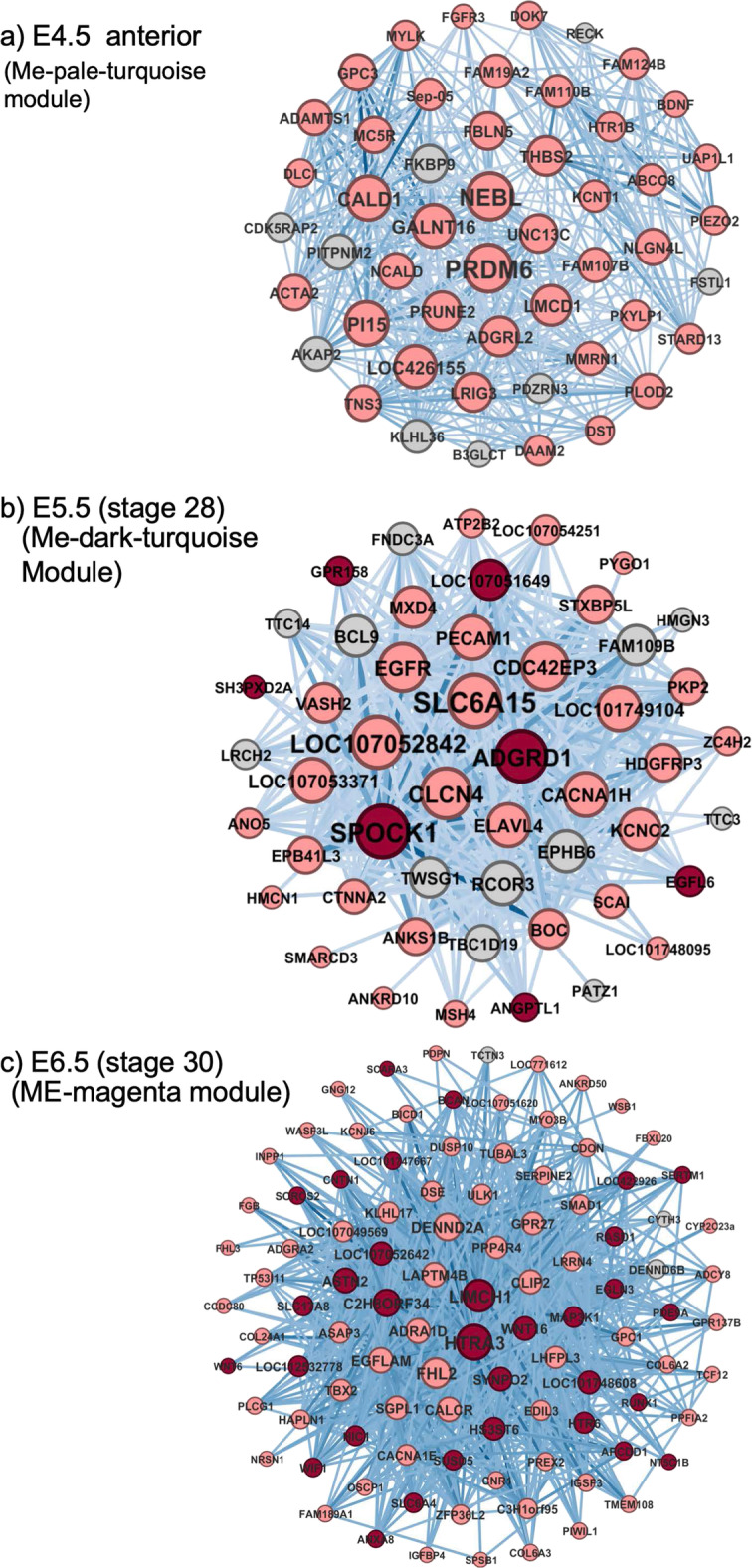
Gene networks of the top enriched modules at each stage of duct formation. Networks were visualized using top 50 or 100 highly connected genes. Colour of nodes show their DEG status. Pink nodes are statically DEGs (compared to stage 4.5 posterior control) and red nodes are DEGs in both static and dynamic comparisons. For dynamic changes it has been compared with the previous stages except for E4.5-Ant. (compared to 4.5 posterior stage). Grey nodes are non-DEGs. Larger nodes have higher degree of “hubness”. Only 500 or 1000 highly weighted edges are shown. Thicker edges (darker colour) represent higher weight

WGCNA applies network-based methods to identify groups of co-expressed genes. Network centrality analysis can be used to detect “hub genes”: those highly connected members to others (see Methods section). We visualized and analysed candidate modules to detect hub genes using weighted degree centrality analysis. This detects how many nodes are connected to the node of interest by correlated expression. We selected the top 50 or 100 nodes with highest degree or relatedness. DEGs in each module were detected to show whether the hub genes were also showed changes in expression compare to other stages. We found that some of the hub genes were differentially expressed (DEG) through Mullerian duct development, while other hub genes were not differentially expressed. Here, we focus on the DEGs in the modules. The most significant gene network at each stage of Müllerian duct development is shown in Fig. 3. In the E4.5 anterior duct, the pale-turquoise module had several DEGs. Most members of this module were DEGs. *NEBL, PRDM6, CALD1, PI15, GALNT16, LOC426155, LMCD1, ADGRD2, LRIG3*, and *PRUNE2* were the top 10 differentially expressed hub genes at this developmental stage (Fig. 3a). For 5.5 duct (in which tissue was duct only, with no mesonephric kidney), most of the genes in the dark-turquoise module were DEGs. *SLC6A15, LOC107052842, CLCN4, SPOCK1, ADGRD1, EGFR, CDC42EP3,* and *CACNA1H* genes were all DEGs compared to E4.5 posterior control stage. Only *SPOCK1, ADGRD1* genes are also up-regulated dynamically (compared to the earlier 4.5 anterior tissue) (Fig. 3b).

For the 6.5 duct stage, almost all the members of magenta module were DEGs, statically (compared to E4.5 posterior) or dynamically (compared to each other) (Fig. 3c). *HTRA3, LIMCH1, C2H8ORF34, ASTN2, WNT16,* and *SYNPO2* were all DEGs statically or dynamically, whereas, *FHL2, DENND2A, EGFLAM, ADRA1D, LAPTM4B, CALCR,* and *CLIP2* were only statically DEGs (compared to the E4.5-Post control). Known Mullerian genes such as *WNT4* and the *HOX* genes were present in the gene networks, but were not among the top 50–100 hub genes in the network. Some of these hub genes were tested by in situ hybridization, for example *HTRA3* was highly expressed in the ME-Magenta hub at E6.5 (Supplementary Figure 5). In addition to strong expression is a region of the mesonephros, *HTRA3* was expressed in the Müllerian duct mesenchyme. It encodes a serine protease that cleaves extracellular matrix proteoglycans and also inhibits TGF-β growth factor signaling [44].

### Transcription factor analysis

**Fig. 4:**
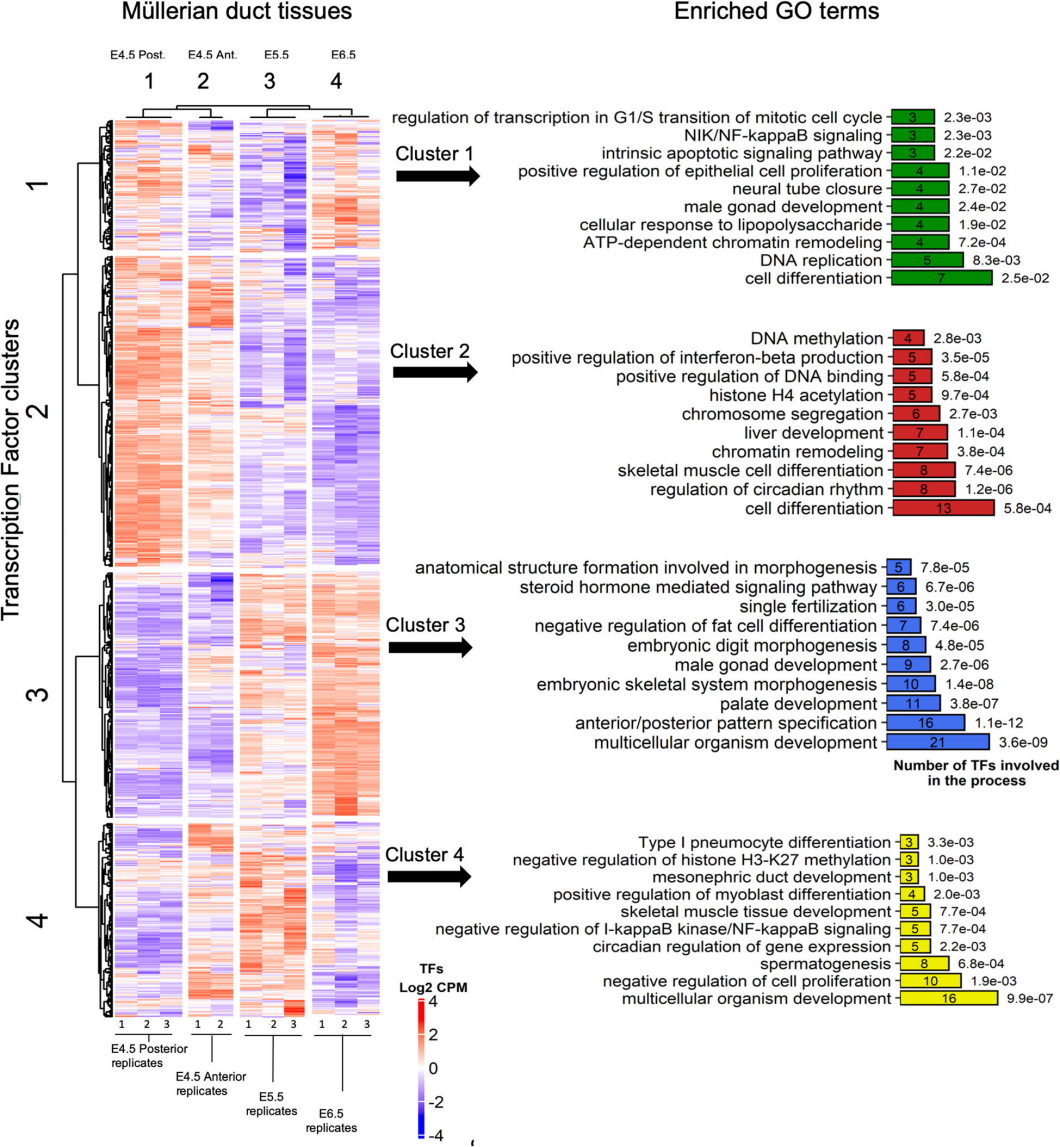
Heatmap plot showing clustering of all transcription factors expressed during chicken Mullerian duct development and GO of their members. Different colours are related to each cluster. Key of the heatmap is after scaling the CPM; red = highly expressed, blue = lowly expressed. Numbers beside each bar represent *P*-values

In this analysis, we focussed on transcription factors, given their essential role as core developmental regulators. The datasets were analysed in different ways to identify important new transcription factors (TFs) throughout development of the Mullerian duct. Through PPI network analysis we could not find any cluster for TFs as information for PPI interaction of TFs is limited. Therefore, we used hierarchical clustering of all genes encoding a protein with GO terms “DNA-binding” or “transcription factor activity”. Genes related to non-TF functions were manually removed, yielding a final list of 1212 known and potential TF genes. We performed Elbow analysis to determine the number of clusters (k number) for grouping samples and genes. After hierarchical clustering-based on correlation distance, genes could be bioinformatically clustered into 4 broad groups. As expected, the replicates of each stage could be transcriptionally grouped into 4 clusters. Figure 4 shows a transcription factor heat map of the four broad clusters across the four tissue groups (E4.5 posterior, E4.5 anterior, E5.5 and E6.5.) (Only two replicates for E4.5 anterior tissue, as noted previously.) In terms of transcription factor expression, it can be seen on the heat map that the E4.5 posterior and E4.5 anterior samples cluster together (as expected, because they share mesonephric kidney tissues), while the E5.5 and E6.5 samples (comprising Mullerian duct only) cluster together. GO analysis was performed for members of each cluster and results were filtered to remove any non-specific TF’s terms, such as “Transcription factor activity”, “regulation of transcription”, “DNA binding”. Finally, a list was compiled of enriched biological processes linked to the transcription factors highly expressed in each cluster.

Cluster 1 comprised a small cluster of TFs that were highly expressed at the E4.5 posterior samples and in the E6.5 duct, but more lowly expressed in other stages. Enriched GO terms were linked to cell differentiation, DNA replication, and chromatin remodelling (Fig. 4, green). Cluster 2 encompassed the greatest number of grouped TFs, expressed at E4.5 posterior (control) and E4.5 anterior stages and lowly expressed in tissues comprising Müllerian duct only (E5.5 and E6.5). As both the E4.5 tissues (anterior and posterior) comprised mainly mesonephros, this cluster is inferred to be primarily linked to mesonephric kidney development. Enriched TFs were those linked to cell differentiation, organ differentiation, chromatin remodelling and histone modification (Fig. 4, red). Cluster 3 was specifically associated with duct development, being progressively up-regulated in E5.5 and E6.5 ducts relative to E4.5. Transcription factor GO terms enriched in this cluster included those related to multicellular organism development, anterior/posterior pattern formation, palate development, and embryonic skeletal system morphogenesis (Fig. 4; blue). This important cluster included transcription factors already known to be important for duct Müllerian formation, such as *DMRT1, HOXA10* and *HOXD10*. Interestingly, cluster 4 was highly expressed in the E5.5 ducts (invagination and early elongation). Enriched GO terms at this stage were those related to multicellular organism development, negative regulation of cell proliferation, spermatogenesis, regulation of circadian gene expression, NF-κB signaling and skeletal muscle development (Fig. 4, yellow).

### Validation of gene expression in developing Müllerian ducts

**Fig. 5:**
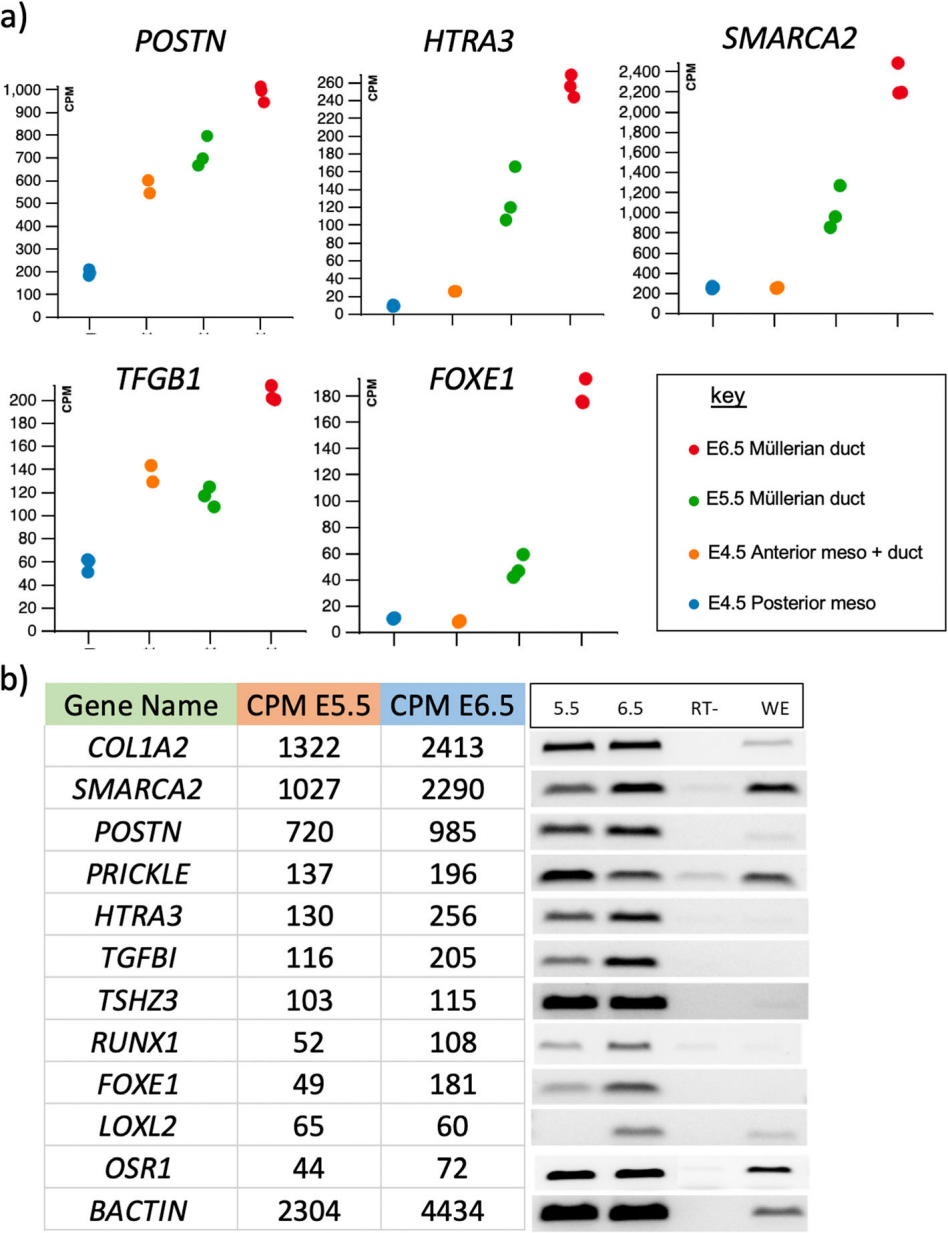
Expression of top genes differentially expressed during embryonic Müllerian duct formation (2 > log2 FC; with cpm > 50 by E6.5). **a** Expression from the RNA-seq data for the top five DE genes, enriched over duct development (cpm). **b** RT-PCR analysis of gene expression in isolated ducts over E5.5–6.5. Detection of mRNA transcripts of *COL1A2*, *SMARCA2*, *POSTN*, *PRICKLE*, *TSHZ3*, *HTRA3*, *TGFBI*, *RUNX1, FOXE1*, *LOXL2* and *OSR1*. (RT- = no RT enzyme; WE = whole embryo at E4.5). Figure shows cropped gel images for clarity. (Un-cropped gel images shown in Supplementary Figure 6)

Novel top differentially expressed genes in the developing Müllerian duct were chosen for validation (those with high cpm > 50, with 2 > log2 fold expression at E6.5 change relative to E4.5 posterior mesonephros control). These genes were highly up-regulated during duct formation in the RNA-seq dataset (lowly expressed or not expressed in the E4.5 posterior mesonephric kidney but up-regulated in developing ducts) (Fig. 5a). These data were validated by RT-PCR, conducted on stage matched tissues at E6.0 (HH stage 31). Figure 5b shows the RT-PCR data at E5.5 (stage 28) and E6.5 (stage 30), for the top differentially expressed genes. These genes encode cell adhesion or matrix proteins (*POSTN, COL1A2, TFGBI)*, matrix re-modelling enzymes (*LOXL2, HTRA3*,) and novel transcription factors or chromatin modifiers (*TSHZ3*, *FOXE1*, *OSR1*, *SMARCA2, PRICKLE* and *RUNX1)*. We short-listed genes for further mRNA in situ hybridization analysis based on these criteria; transcription factors and signaling molecules, those with reported function and involvement in cell proliferation or migration, and those previously not linked to Müllerian duct formation. These genes represented novel candidates involved in embryonic Müllerian duct formation.

**Fig. 6:**
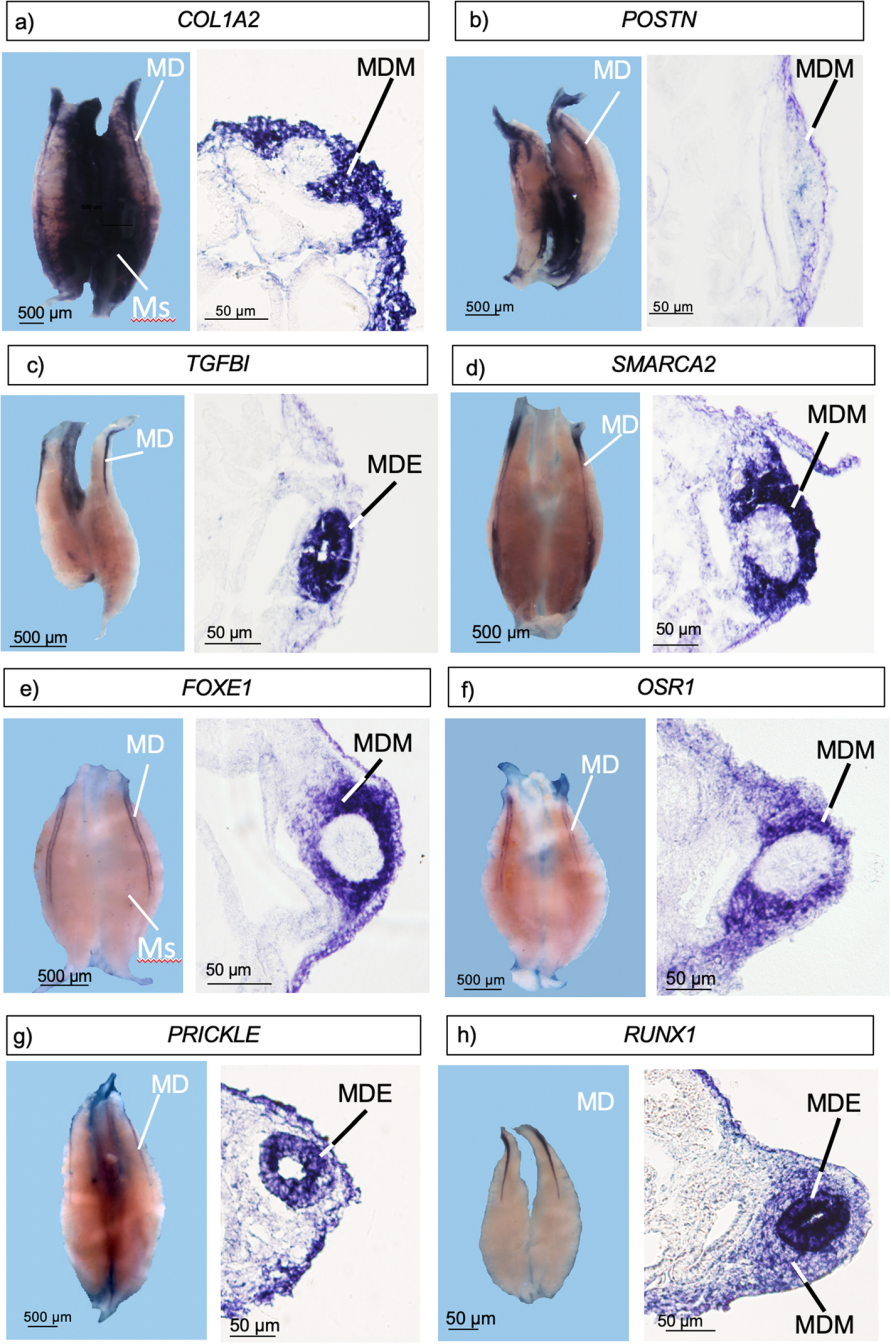
Whole mount in situ hybridization and sectioned whole mounts for candidate duct genes, in E6 (stage 29) urogenital systems (UGS). **a** COLA2, widely expressed in the UGS and in duct mesenchyme (MDM). **b** *POSTN*, localised in the mesenchyme. **c**, *TGFBI*, strongly expressed in the Müllerian duct epithelium (MDE). **d** *SMARCA2*, strongly expressed in the duct mesenchyme (MDM). **e** *FOXE1*, expressed in the duct mesenchyme (MDM). **f** *OSR1*, expressed in duct mesenchyme (MDM). **g** *PRICKLE*, expressed in the duct epithelium (MDE). **h**. RUNX1, strongly expressed in the duct epithelium (MDE) and also in the mesenchyme (MDM)

The RT-PCR results showed differential gene expression in E5.5 and E6.5 Müllerian ducts. Whole-mount in situ hybridization (WISH) analysis of the candidate genes was performed on embryonic chicken Müllerian duct at day 6.0 of incubation (stage 28), the mid-point between E5.5 and E6.5. Following whole mount staining, urogenital systems were sectioned and examined for expression within the tissue. All of the top differentially expressed genes in the RNA-seq datasets were validated by whole mount in situ hybridization (Fig. 6). The cell adhesion or matrix genes, *COL1A2, POSTN, TFGBI,* were all strongly expressed in developing ducts, and also in parts of the interstitium between the paired mesonephric kidneys. *POSTN* and *COL1A2* were expressed in the Müllerian duct mesenchyme (MDM), while *TFGBI* mRNA localised to the inner Müllerian duct epithelium (MDE) (Fig. 6a, b and c). Among the novel highly expressed transcription factor or chromatin modifier genes identified during duct development, *SMARCA2, FOXE1* and *OSR1* were all expressed in the duct mesenchyme (Fig. 6d, e and f). *SMARCA2* showed stronger expression at the two poles of the duct (Fig. 6). *PRICKLE* mRNA was confined to the Müllerian duct epithelium (Fig. 6g), while *RUNX1* was strongly expressed in the epithelium, but also expressed in the mesenchyme (Fig. 6h).

### *FOXE1* expression in the embryonic chicken Müllerian duct

**Fig. 7:**
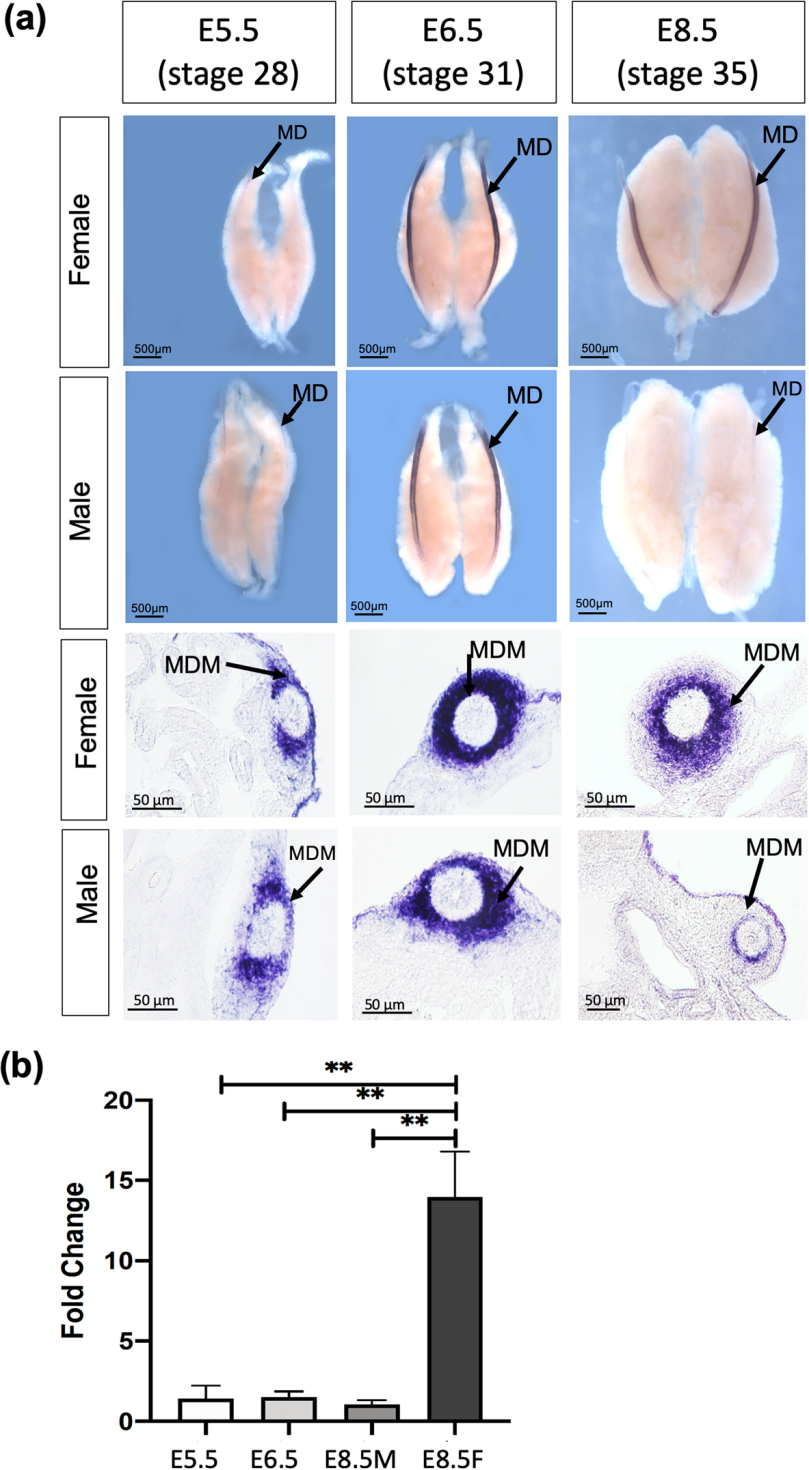
Expression profile of *FOXE1* mRNA in the embryonic chicken Müllerian duct. **a** Time course of *FOXE1* mRNA expression in the male and female Müllerian duct during development, as assayed by whole mount in situ hybridization and sectioned whole mounts. Expression is first detected in female and male Müllerian duct at E5.5 (stage 28) and 6.5 (stage 30). At E8.5 (stage 34), *FOXE1* is strongly expressed in the developing female ducts, but weak staining is detected in male ducts. Expression is not detected in gonads and mesonephros. Sectioned whole mounts (upper images) show expression of *FOXE1* detectable throughout the developmental stages in Müllerian duct mesenchyme (MDM), with no expression in Müllerian duct epithelium (MDE). At E8.5 (stage 34), *FOXE1* is strongly expressed in the developing female ducts but weak expression in male ducts. **b** Quantitative RT-PCR analysis of *FOXE1* mRNA expression in E5.5, 6.5 and E8.5 male and female Müllerian ducts. Expression levels were normalized to *β-ACTIN* and expressed relative to E5.5. Bars represent Mean ± SEM. ** = adjusted *p* value< 0.01. One-way ANOVA and Tukey’s multiple comparisons test

One of the novel transcription factor genes strongly up-regulated during duct formation was *FOXE1*. Given the importance of FOX transcription factors in the female urogenital development [45, 46], this gene was further studied. Based on in situ hybridization, *FOXE1* transcripts were not detected in E4.5 (stage 25) (not shown) but the onset of expression was detected in E5.5 (stage 28) ducts of both sexes. This corresponds the duct elongation phase. Expression was strong at E6.5 and it persisted in female ducts developing at E8.5 (stage 34). In males, expression was lost by E8.5, coincident with the onset of duct regression (Fig. 7a). Sectioned whole mounts showed that mesenchymal expression was maintained in female ducts as they developed, but was down-regulated in male mesenchyme. Quantitative RT-PCR, confirmed significant up-regulation of *FOXE1* in female but not male ducts at E8.5 (stage 34) (Fig. 7b).

### Long non-coding RNAs expressed during Müllerian duct formation

**Fig. 8:**
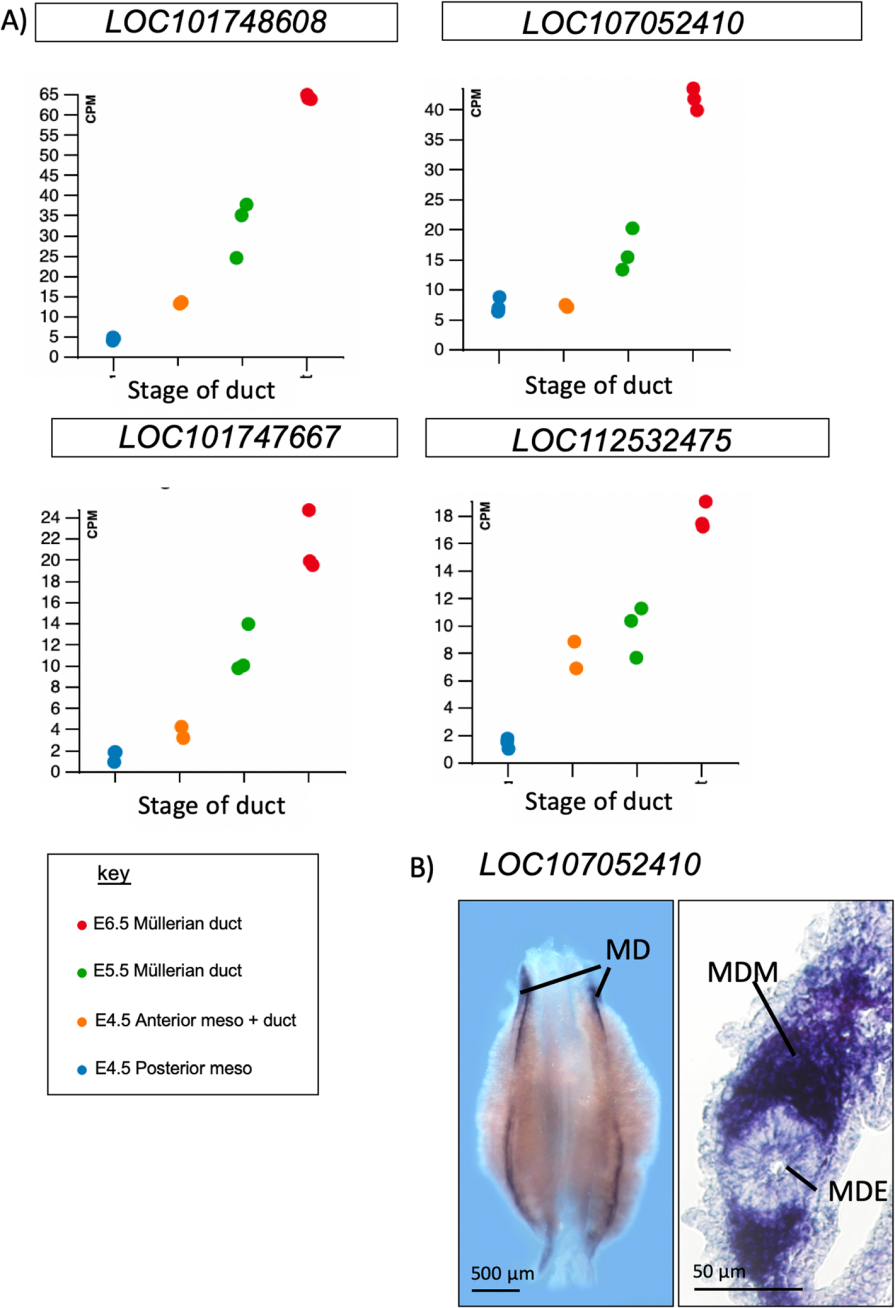
Expression of lncRNAs in chicken Müllerian ducts. Top differentially expressed lncRNAs are shown. **a** RNA-seq data, showing increasing expression of three uncharacterised lncRNAs during duct formation. **b** In situ hybridization analysis of lncRNA *LOC107052410*.This non-coding RNA shows polarised expression E6.0 (stage 29) Müllerian ducts, localised to the duct mesenchyme. (MDM)

Long non-coding RNAs (lncRNAs) were prevalent in the Müllerian duct datasets. One thousand two hundred seventy lncRNAs were detected, suggesting that non-coding RNAs play an important role in Müllerian duct formation. Of these transcripts, 132 lncRNAs showed a log2 fold change at an FDR cut off of 0.1. Based on these criteria, the four top differentially expressed lncRNAs are shown in Fig. 8a. One of these, a Z-linked non-coding RNA (*LOC107052410*) was examined by in situ hybridization. This confirmed expression in the mesenchyme of the developing Müllerian duct (Fig. 8b). Most lncRNAs in the datasets showed an increase in expression during duct development. We did not detect any lncRNAs that were enriched specifically at the earliest stage (E4.5). Interestingly, one of the lncRNAs identified was *HOX10A-AS*, expressed from the anti-sense strand of the *HOX10A* locus, and a known regulator of *HOX10A* [47]. *HOX* genes are important for patterning the Müllerian duct [48] and *HOX10A* mutation are linked to developmental anomalies of the reproductive tract in humans [49].

## Discussion

The molecular genetic pathways regulating Müllerian duct formation are incompletely understood. The study reported here sheds new light on this process by describing for the first time, in any species, the transcriptome of the developing Müllerian duct. The datasets reveal novel genes and developmental pathways that are activated during duct formation, providing a rich source of genetic data for further research into the molecular control duct morphogenesis. One previous study carried out RNA-seq on the mesenchymal compartment of mouse Müllerian duct specifically during the phase of duct regression in male embryos [50]. The aim of that study was to identify genes and pathways engaged by AMH during the regression process. Eighty two genes were up-regulated in male mouse duct mesenchyme during AMH signaling. Of these genes, only a small number were also present in our dataset. These were the homeobox transcription factor, *Msx2*, *Ednrb* (endothelin receptor), *Transgelin* (actin-binding protein) and the extra-cellular matrix genes, *Ecm1, Col15a1* and *Col9a3*. This indicates that the genes regulating duct formation are different to those involved in later duct regression in males.

In the developing Müllerian duct of chicken embryos, we identified transcripts of genes previously implicated in duct specification, invagination and elongation (Supplementary Figure 1). In addition, a large number of differentially expressed novel genes were identified. As duct development progressed, an increasing number of genes were up-regulated compared to the E4.5 posterior mesonephros (control in static comparisons). Nine hundred six genes were differentially expressed in ducts compared to the control, while 188 genes showed differential expression dynamically across the three stages of duct development (E4.5 anterior vs E5.5 vs E6.5) (Fig. 1). Gene ontology analysis showed that specific developmental pathways were enriched during duct formation. These included pathways associated with cell adhesion, cell migration, cell proliferation, negative regulation of apoptosis, G-protein coupled receptor signaling, the extracellular matrix and WNT and ERK signaling (Fig. 2). This is consistent with the cellular behaviour that occurs during duct morphogenesis, which involves proliferation, migration, interaction with the extra-cellular matrix, and epithelial-mesenchyme cross-talk [43]. In rodent and chicken embryos, the Müllerian duct elongates through cell proliferation and active migration at the tip of the caudally progressing duct epithelium [23, 29, 39, 51]. Epithelial-matrix interactions appear to be important for this process, as we recently described a requirement for the cell adhesion-linked factor, *GPR56*, in chicken Müllerian duct development [52].

Indeed, Müllerian duct formation is an elegant example of tubulogenesis (the organisation of epithelia into tubular structures) [53]. Epithelial tubes are fundamental structures in biology, making up much of the vascular, respiratory and gastrointestinal systems, as well as the reproductive tract. Tube formation in these other contexts relies heavily upon epithelial-mesenchyme interactions, G-protein coupled signaling and dynamic protein and glycoprotein functions within the extra-cellular matrix [54–56]. These pathways were enriched in the developing avian Müllerian duct (Fig. 2 and Supplementary Figures 2 and 3). In other systems that feature tube formation through invagination, lumen development requires epithelial cell polarisation, dynamic cytoskeletal rearrangements, GTPase and metalloprotease activity [53, 55, 57]. Genes related to these pathways were enriched in our datasets, in both static and dynamic comparisons. In other systems, Receptor Tyrosine Kinase (RTK) pathways play a central role in mediating tubulogenesis [53]. Pathway ligands include FGFs, VEGFs and EGFs. Among these, FGF is required for *Lim1* gene expression and Müllerian duct specification in chicken [21], while PI3 Kinase/AKT mediates duct elongation in rodents [51]. There is likely to be a requirement for RTK signaling in subsequent duct invagination and elongation in chicken, as ERK1 and ERK2 transduction pathways were enriched in our datasets at E5.5 and E6.5 (Fig. 1). *VEGFD* and *FGF-10, − 16* and *− 18* were all up-regulated during duct formation reported here. In the kidney, the RTK ligand Hepatocyte Growth Factor (HGF) has been shown to have tubulogenic properties, promoting cell migration and invasion [58, 59] and we found that this signaling molecule was up-regulated during duct formation in chicken. Similarly, we detected up-regulated components of the TGF-β signaling pathway (TGF-β1, 2 and 3 and their receptors, and GDF-7). TGF-β plays a role during tube formation in the quail cardiovascular system [60]. These comparisons point to conserved pathways regulating tube formation in different developmental contexts.

In the dynamic comparisons of DEG’s, major gene networks that were enriched during duct development included G-protein coupled receptor signaling, cell adhesion, cell migration, focal adhesion, cell polarity, and Fgf and Wnt4 signaling (Supplementary Figures 2 and 3), consistent with previous reports for other types of tubulogenesis [61–64]. Fewer differential networks were evident in E5.5and E6.5 comparisons, when the duct is in the elongation phase (Supplementary Figure 3). At this stage, G-protein coupled receptor signaling, cell adhesion and muscle tissue morphogenesis. Terms related to positive and negative Wnt signaling also featured prominently in the GO analysis reported here. Wnt signaling is a well-established conserved pathway required for proper Müllerian duct formation in chicken, mouse and human [22, 35, 40, 65, 66]. Wnt9b derived from the Wolffian duct is required for inductive signaling to the incipient Müllerian duct [34]. *Wnt4* expression in the duct mesenchyme is essential for the invagination and cell migration during elongation phase of duct development [22, 67]. *Wnt7a* and *Wnt5a* play roles in duct elongation [30, 31], while the former is required for duct regression in male embryos [65]. We identified a number of WNT genes up-regulated during chicken Müllerian duct formation, including *WNT4*, *WNT16, WNT6, WNT7A*, *WNT7B, WNT2* and *WNT10A*. In addition, novel duct genes associated with WNT signaling or its inhibition were identified. These genes were up-regulated in the developing ducts and included *APCDD1*, a membrane-bound WNT inhibitor [68] and the Dickkopf family of WNT signaling inhibitors. Given the importance of Wnt signaling for duct formation, further research into these modulators of the pathway is warranted.

Gene network and pathway analyses revealed a novel link between Müllerian duct formation and neuronal-related pathways, such as neuron cell migration and axonal guidance (Fig. 2, Supplementary Figures 2 and 3). Transcripts enriched in the Müllerian duct that are related to axonal guidance or neuronal migration included *EFNB2* (Ephrin B2), *UNC5D* (Netrin receptor), the guidance factor *SLIT1* and in particular, the class-A semaphorins (*SEMA6D, SEMA3G* and *SEMA5A*), which are chemorepellents [69]. One possibility is that the RNA-seq captured neuronal cells present in the forming ducts. However, innervation of the duct at these early stages has not previously been reported, in chicken, mouse or human. This includes fine ultrastructural studies [20, 21, 39, 70, 71]. Alternatively, these enriched networks suggest that migrating duct epithelial cells may rely upon similar signaling pathways to those of migrating or elongating neurons. During development, neural crest cells migrate by following paths laid down by cell adhesion molecules in the extra-cellular matrix, while neurons of the CNS migrate along trails of glial supporting cells [72]. In terms of axonal guidance, both attractive and repellent signaling factors are known [69]. The presumed chemotactic cue that drives cranio-caudal migration of the Müllerian duct is unknown. This signal/s may be a positive guidance factor secreted by the posterior mesenchyme (or kidney), or it could be a repulsive factor concentrated at the anterior pole. A morphogen gradient is likely to be involved, but classic morphogens such as Hedgehog or WNT do not show the appropriate graded expression pattern [40]. In the developing chicken Müllerian duct, we detected enriched expression of *RGMB,* Repulsive Guidance Molecule BMP Co-Receptor B, which is known to contribute to axonal guidance and regeneration [73, 74]. This factor may also be therefore be important for Müllerian duct elongation. The epithelial tip cells of the elongating Müllerian duct are crucial for proper duct development. Little is known about the exact properties of these cells, although studies have shown that they actively proliferate during duct elongation in the chicken [23]. It would be of interest to culture embryonic Müllerian duct in the presence of some of the neuronal-related factors identified here, to assess effects upon cell migration and proliferation.

The data presented here reveal novel genes implicated in vertebrate Müllerian duct development. The WCNA analysis revealed novel genetic modules engaged during the period of duct development. For example, the pale-turquoise module revealed at 4.5 anterior (i.e. mesonephric kidney plus duct anlagen) (Fig. 4a), comprised genes related to cell adhesion and actin filament organization. These processes are indeed required for the rearrangement of cells to form the invaginating cells of the early chicken Müllerian duct [21]. Other components of these modules included genes with roles in integrin-mediated signaling, as expected of cells invaginating and giving rise to duct mesenchyme via EMT (Supplementary Table 2). We identified several transcripts that were enriched specifically in these E4.5 anterior tissues and not at later stages. These represent novel candidate regulators or duct specification and invagination. They included *GALNT16* (a glycosylating enzyme), *SEMA3G* (a cell migration and invasion-linked semaphorin), *ADRB2* (adenoreceptor-beta 2) and *CBLN1* (cerebellin-precursor, required for neuronal synapse function). *THSB2* (Thrombospondin-2) was highly up-regulated specifically in E4.5 anterior tissues (the stage of duct invagination). Thrombospondins play decisive roles in cell-cell and cell-matrix interactions [75].

Focussing on the ducts alone at E5.5 and E6.5, WCNA analysis revealed novel hub genes that showed high levels of expression in both static and dynamic comparisons and high-level connectivity to other genes. These included *ADGRD1* and *SPOCK1*at E5.5 and *LIMHC1* and *HRTA3* at E6.5. *ADGRD1* encodes a G-protein coupled receptor of the D sub-family (GPR133). G-protein coupled receptors were prominent in our datasets, pointing to a pervasive role in Müllerian duct formation. These large membrane-bound proteins transduce extra-cellular matrix signals through G proteins [76]. We recently demonstrated a requirement for one such family member, GPR56, in the chicken Müllerian duct [52]. *SPOCK1* (testican) also has a matrix association, encoding proteoglycan containing chondroitin- and heparan-sulfate chains, with unclear function [77]. *LIMCH1* encodes a protein that positively regulates actin stress fibre assembly and negatively regulates cell spreading and cell migration [78]. HTRA3 is a serine protease that cleaves ECM-associated proteoglycans. It also inhibits TGF-β signaling and regulates cell invasion [79–81]. Here, we found the gene to be expressed in the duct mesenchyme (Supplementary Figure 5). Altogether, the data indicate that these structural, cell adhesion and signal transduction factors are central hubs for Müllerian duct development.

More detailed expression analysis was conducted on a short list of differentially expressed genes (Figs. 5, 6 and 7). These genes were either very highly differentially expressed in the duct and/or encoded transcription factors. Transcription factors were prioritised due to their leading role as developmental regulators. Among these highly expressed genes were the transcription factors, *RUNX1, OSR1, PRICKLE* and *FOXE1.* Previous studies link *RUNX1* to the female reproductive tract*.* Runx1 has a role in the mouse ovary, where it maintains the fetal granulosa cell identity through interaction with Foxl2 [82]. Conditional deletion of *Runx1*in the embryonic mouse Müllerian duct results in failure of regional reproductive tract differentiation into vaginal epithelium postnatally [83]. The data presented here suggest that *Runx1* may also have an earlier critical role in duct formation, where it is expressed in both epithelium and mesenchyme (Fig. 6h). The odd-skipped-related transcription factor, *Osr1*, is a key driver of intermediate mesoderm formation and urogenital development [84, 85]. *Osr1*^*−/−*^ mice have complete agenesis of the adrenal glands, metanephric kidneys and gonads [84]. This early profound phenotype has precluded any analysis of the developing Müllerian ducts. The data presented here suggest a novel role for *Osr1* in the mesenchymal compartment during Müllerian duct formation.

*PRICKLE1* was strongly up-regulated during duct formation. This gene encodes a nuclear receptor that localises to the nuclear membrane and is implicated in planar cell polarity, inhibition of the Wnt/β-catenin signaling pathway, EMT and neuronal cell migration [86]. In the chicken embryo, *PRICKLE1* is expressed in a developmentally restricted fashion, in the primitive streak, ventral neural tube, and foregut at early stages and later in differentiating myotomes [87]. This is the first report of duct expression for *PRICKLE1*. Another mRNA highly enriched in the developing Müllerian duct was the forkhead box transcription factor, *FOXE1* (Fig. 7e, Fig. 1). Another well-characterised forkhead box gene, *FOXL2*, is required for ovarian development in chicken and other vertebrates [46, 88–91], while *FOXD1* was recently implicated in chicken testis development [92]. *FOXE1* has not previously been linked to development of the reproductive tract in any species. Here, we found that the gene is strongly expressed in the duct mesenchyme during development, and is down-regulated in males during duct regression (Fig. 7). In other tissues, *FOXE1* operates as a tumor suppressor [93–95]. Recent studies have shown that FOXE1 regulates cell proliferation and migration by activating the Wnt/β-catenin pathway in thyroid carcinoma cells [96, 97]. *FOXE1* mutations in humans cause Bamforth-Lazarus syndrome, characterized by thyroid and craniofacial defects, although no reproductive tract abnormalities have been reported [98]. *Msx1* and *Tgf-β3* are reported targets of Foxe1 in the developing palate [99]. We postulate that *FOXE1* in the chicken Müllerian duct regulates cell migration and/or EMT, given its mesenchymal localisation.

Other highly up-regulated genes in our data were *POSTN,* a secreted extracellular matrix protein that binds integrins to support cell adhesion and migration, *SMARCA2,* a transcriptional regulator via chromatin modification, and *TGFBI,* another extracellular matrix protein, which modulates cell adhesion and serves as a ligand recognition sequence for several integrins (Fig. 6). *TGFBI* plays a role in cell-collagen interactions, inhibition of cell adhesion and is induced by TGF-β signaling [44, 100–106]. Up regulation of *TGFBI* has been reported in a broad variety of diseases, including corneal disorders, nephropathy [107], rheumatoid arthritis [108], cancer [102, 104, 109] and atherosclerosis [110]. Lastly, our data reveal a novel involvement of long non-coding RNAs in formation of the Müllerian duct. Long non-coding RNAs have been implicated in phallus development in the wallaby [111], but this is the first report of lncRNA expression in the embryonic female reproductive tract of any species. A large number of lncRNAs were identified in this study. Their role in the Müllerian duct is speculative, but lncRNAs in other tissues can have a range of functions, from chromatin modification and transcriptional regulation in the nucleus through to translational regulation and microRNA modulation in the cytoplasm [112].

## Conclusions

In summary the data presented here provide a detailed view of the developmental pathways engaged during vertebrate Müllerian duct development. A number of novel genes and pathways have been revealed, notably those involved in Wnt, MAP kinase and TGF-β signaling, cell migration factors and factors interacting with the extra-cellular matrix. Due to its development *in ovo* and hence ease of experimental manipulation, the chicken model is ideal for live cell studies and gene manipulation during duct formation. For example, Atsuta et al. (2013) used *in ovo* electroporation and time lapse imaging to label Wolffian duct precursors with EGFP in the chicken embryo, allowing the tracking of migrating cells. Wolffian duct elongation was aberrant when a Rho inhibitor electroporated [113]. A similar approach could be used to manipulate factors and then observe cell behaviour during Müllerian duct elongation. We and others have used *in ovo* electroporation to manipulate endogenous gene expression in the embryonic chicken Müllerian duct [21, 23, 40]. TOL2 transposons that integrate into the genome can be electroporated into the nascent duct, delivering transgene over-expression or shRNA-mediated knockdown [52]. These approaches can be used for rapid functional analysis of the novel genes and pathways identified here. The data also provide a transcriptomic resource for research on other models of tubulogenesis.

## Methods

### Embryo incubation and tissue collection

Fertilized eggs of Hy-Line Brown chickens (*Gallus Gallus domesticus*) were purchased from Hy-Line Australia, Pty Ltd., Woodend, Victoria, and incubated at 37.8 °C with rocking under humid conditions. For RNA-seq, embryos were harvested at three key developmental stages of Müllerian duct formation: specification and invagination (embryonic day (E) 4.5/HH stage 25), duct elongation (E5.5/HH stage 28 and E6.5/HH stage 30), [114].

### RNA extraction, library preparation and RNA sequencing

Total RNA was extracted from pooled embryonic chicken urogenital systems and Müllerian ducts at embryonic day (E) 4.5, E5.5 and E6.5. These stages cover the non-sexually dimorphic phase of duct formation in chicken. Tissues were therefore not separated by sex. These stages also pre-date the later regression phase of duct development (after E7.5), in which both ducts regress in males and the right duct regresses in females. This project focussed on duct formation rather than regression. Due to the difficulty of cleanly dissecting nascent Müllerian duct at E4.5, the entire anterior third of the paired urogenital system was taken. This comprised the anterior portion of the paired mesonephric kidneys, some attached midline mesentery and the region of coelomic epithelium that undergoes specification and invagination at the onset of Mullerian duct formation (also called the Müllerian ridge). The posterior two thirds of the urogenital system served as negative control tissue, comprising mesonephros and mesentery but no Müllerian ridge. At E5.5 and E6.5 (HH28 and 30), the developing Müllerian duct could be dissected away from the mesonepehros. The entire duct, including surface epithelium, underlying duct mesenchyme and the (innermost) Müllerian epithelium, was dissected from the adjoining mesonephros as one structure. This was done for each pair of ducts. Tissues samples were collected and pooled in triplicate and comprised in total: 5 paired E4.5 (HH25) anterior urogenital system and E4.5 (HH25) posterior urogenital system negative control × 3 (i.e. left and right urogenital systems from 15 individuals), 3 paired E5.5 (HH28) Müllerian duct × 3 (i.e. left and right Müllerian duct from 9 individuals), 4 paired E6.5 (HH31) Müllerian ducts × 3 (i.e. left and right Müllerian duct from 12 individuals). Pooled tissues for each replicate were snap frozen on dry ice and stored at − 80 °C.

Total RNA was extracted from paired E4.5 (HH25) anterior urogenital system, E4.5 (HH25) posterior urogenital system, E5.5 (HH28) and E6.5 (HH30) Müllerian duct using TRIzol, as per the manufacturer’s instructions. All RNA samples were treated with DNase I using the Ambion DNA-free kit to remove contaminating genomic DNA. After RNA quantification and quality control for RNA integrity, libraries were prepared in triplicate from 300 ng of total RNA using the Illumina TruSeq Stranded mRNA kit using single indexing according to the manufacturer’s recommendation. Barcoded libraries were pooled and sequenced in a single lane of an Illumina NextSeq500 with a V2 75c sequencing kit according to the manufacturer’s instructions at Micromon - Next-Generation Sequencing & Genomics Facility in Monash University, Clayton, Melbourne.

### Bioinformatic analysis

Raw fastq files have been analysed with RNAsik pipeline (Tsyganov et al. 2018) to produce raw genes count matrix and various quality control metrics. For this analysis RNAsik pipeline (Tsyganov et al. 2018) ran with STAR aligner option [115] and reads were quantified with featureCounts [116]. Chicken reference GFF and FASTA files were downloaded from RefSeq database (https://www.ncbi.nlm.nih.gov/assembly/GCF_000002315.6). Raw counts were then analysed with Degust (Powell 2015) web tool to do differential expression analysis to produce list of differentially expressed genes and several quality plots including classical multidimensional scaling (MDS) and MA plots. In this analysis limma voom [42] was used for differential expression analysis. Degust (Powell 2015) largely followed limma voom workflow with typical counts per million (CPM) library size normalisation and trimmed mean of M values (TMM) normalisation [117] for RNA composition normalisation. Differentially Expressed Genes (DEGs) were selected by meeting cut-offs of FDR ≤ 0.05, and Log2FC ≤ 0.585 or ≥ 0.585.

### Network analysis

Protein-Protein Interaction (PPI) networks were constructed by STRING [118] and visualized by Cytoscape 3.7.1 [119]. Only interactions from experiments or databases options with a high confidence (≥ 0.7) were used. To find subnetworks, we applied clustering with Overlapping Neighborhood Expansion (ONE) algorithm using application Cluster ONE 1.0 [120] from Cytoscape. Parameters for finding subnetworks were as follows. Minimum size 5, Minimum density Auto, edge weight was STRING confidence value, and *p*-value ≤0.001. To detected co-expressed genes and their networks relating to each developmental stage, we applied Weighted Gene Co-expression Network Analysis (WGCNA) on all the samples using R programming [121]. Briefly, to remove noise from the data, a matrix of count per million reads (CPM) were filtered for any gene with average CPM of more than 5 and normalized with Log2. The matrix had 11,776 genes that met the cut-offs. A signed network was constructed by a soft-power of 26. All other options remained as default. Module eigengenes highly correlated with each developmental stage were selected for further analysis. For each module, hub genes, as central members of the network, were detected as genes with high weighted degree score. The top 50 or 100 hub genes were presented for each module and visualized by Gephi 0.9.2. Gene Ontology (GO) analysis was performed only on module members with a *p*-value ≤0.05, either for gene significance or module membership scores.

#### Hierarchical clustering and correlation analysis

To perform hierarchical clustering, we applied *k*-means clustering. Appropriate *k* values were detected by Elbow method [122] and distances were calculated with either correlation or Euclidean methods. For correlation analysis we used Pearson’s correlation. All analysis was performed using R programming. As there is no published list for chicken transcription factors, we obtained a list of genes with a Gene ontology related to “DNA binding” or “transcription factor activity”. After filtering the list of genes to exclude any non-transcriptional processes such as DNA repair, helicase activity, etc., a list of 1212 unique genes was obtained. Using this list, based CPM, we clustered TFs in groups through development of the Müllerian duct, based on CPM. Additionally, coordinate plots were provided to show correlated TFs expression.

### Gene ontology

Gene Ontology (GO) analysis was performed using DAVID online tool [123]. We used the top 10, 5 or 3 biological processes with a *p*-value < 0.05 and highest number of genes involved in the process. For GO analysis of DEGs, we used network-based analysis according to similarity to find the most connected processes according to common genes among them. We applied Enrichment Map application from Cystoscope with an edge cut-off similarity ≥0.2 and *p*-value ≤0.05 [124].

### RT PCR and qRT-PCR

Total RNA samples were treated with DNase I using the Ambion DNA-free kit to remove contaminating genomic DNA. Five micrograms of total RNA were reversed transcribed into cDNA using random hexamers using AMV reverse transcriptase (Promega, Cat. # M9004). For RT-PCR, gene-specific primers were used as shown in Supplementary Table 1. RT-PCR was performed using GoTaq Flexi DNA polymerase according to the manufacturer’s instructions (Promega # M8291). PCR cycling conditions were 95 °C × 10 min, (95°c × 30 s; 5 °C × 30 s; 60 °C × 30 s; 72 °C × 30 s) × 32, 72 °C × 5 min, 4 °C hold. Quantitative RT-PCR was performed on triplicate samples using QuantiNova SYBR® Green PCR Kit, as described previously (Hirst et al. 2017). Data was normalised against *β-ACTIN* expression using the Pfaffl method [125]. Data was analyzed using one-way ANOVA followed by Tukey HSD post hoc test (GraphPad Prism). For both RT-PCR and qRT-PCR, control cDNA samples were not incubated with reverse transcriptase.

### Embryo sexing and whole-mount in situ hybridization

For genetic sexing of embryos, a small piece of limb tissue was digested in PCR compatible Proteinase-K buffer and the genomic DNA was used for rapid PCR sexing. By this method, only females show a W-linked repetitive element of the *Xho*I class [126]. For in situ hybridization, chicken urogenital systems were dissected from sexed embryos over stages of development; E4.5 (HH25), E5.5 (HH28), E6.5 (HH30) and E8.5 (HH34). At least three embryos were used for each sex and time point.

Whole mount in situ hybridization was conducted as described previously [127]. To generate RNA probes, the relevant cDNAs were firstly cloned into the pGEM-T Easy vector and confirmed by sequencing. Supplementary Table 1 lists the PCR primers used to generate cDNA’s for in situ hybridization. M13 Forward and Reverse primers were used to amplify the cDNA (= riboprobe template). For antisense and sense (negative control), digoxigenin-labeled RNA probes were generated using the relevant T7 or SP6 RNA polymerase sites present in the amplified PCR product. 300 ng of riboprobe template was used for each reaction. Following synthesis, riboprobes were precipitated in 100% ethanol at − 20 °C and resuspended in 100 μL RNase-free water prior to addition to (pre)-hybridization mix. Whole embryonic urogenital tissues were fixed overnight at 4 °C in 4% paraformaldehyde, pooled by sex and dehydrated into l00% methanol prior to further processing. Briefly, following rehydration of embryos from 100% methanol to PBTX (PBS + 0.1% Triton X-100), tissues were treated with Proteinase K in PBTX (10μg/mL) for 30–90 min, depending on their age. Tissues were briefly re-fixed in PFA and equilibrated overnight in hybridization buffer at 65 °C. RNA probes tissues were then added overnight at 65 °C. Tissues were then washed at low and high stringency washes to remove unbound, pre-blocked with in Tris buffer (pH 7.5) containing triton- 100 containing 5% sheep serum and 2% bovine serum albumin (BSA). Tissues were then incubated overnight with an anti-digoxigenin-alkaline phosphatase conjugated antibody (1:2000; Roche). Samples were then washed TBTX, equilibrated in NTMT buffer and developed in the dark with chromogen (BCIP/NBT in Tris buffer, pH 9.5). Tissues were imaged using Leica WILD MZ8 - Leica DC300 dissecting light microscope. Tissues were then washed in TBTX (10 min), NTMT (10 min) and over-stained in NTMT + BCIP/NBT at room temperature over 2–3 days. Tissues were then processed for cryo-sectioning and frozen sections were cut at 14 to18 μm and mounted onto slides for imaging.

## Supplementary information

**Additional file 1: Supplementary Figure 1.** Heatmap plot, showing expression of known genes implicated in the stages of Müllerian duct development (specification, invagination, elongation and differentiation) (Log2 cpm). Transcripts of known genes such as *PAX2, LIM1 (LHX1), WNT4, DMRT1* were all present and enriched in the datasets.

**Additional file 2: Supplementary Figure 2.** PPI network analysis and Gene ontology of the clusters (sub-networks) for static comparison of samples.

**Additional file 3: Supplementary Figure 3**. PPI network analysis and Gene ontology of the clusters (sub-networks) for dynamic comparison of samples.

**Additional file 4: Supplementary Figure 4.** Weighted Gene Co-expression Network Analysis. a) Hierarchical clustering of gene expression groups of individual samples by developmental stage. b) Clustering and merging modules with 75% similarity. c) Module heatmap. Expression 18 colour-coded modules (Red = high level expression, green = low level expression). Some modules positively correlated with duct development (e.g, magenta, highlighted), while others were negatively correlated (e.g., dark olive green). Post_4.5 = negative control tissue. d) Gene significance vs module membership plots of positively correlated modules with each stage.

**Additional file 5: Supplementary Figure 5.** Expression of a selected hub gene, *HTRA3*, in chicken Müllerian duct. The gene is expressed in the Müllerian ducts and in the anterior region of the mesonephros. Sectioned whole mount in situ’s showed expression in the mesenchymal compartment.

**Additional file 6: Supplementary Figure 6.** RT-PCR analysis. Un-cropped gel images of RT-PCR analysis shown in Fig. 5b.

**Additional file 7.**

**Additional file 8.**

## Data Availability

The raw RNA-seq data described in this paper has been deposited on the NCBI Geo portal (GEO ID - Series GSE153029). https://www.ncbi.nlm.nih.gov/geo/query/acc.cgi?acc=GSE153029. The data show the average cpm for each gene and log2fold change.

## Abbreviations

*ADGRD1*: Adhesion G Protein-Coupled Receptor D1
*ADRA1D*: Adrenoceptor Alpha 1D
*APCCD1*: Adenomatosis Polyposis Coli Down-Regulated 1
*ASTN2*: Astrotactin 2
*BMP*: Bone Morphogenetic Protein
*CACNA1H*: Calcium Voltage-Gated Channel Subunit Alpha1 H
*CALCR*: Calcitonin Receptor
*CALD1*: Caldesmon 1
*CBLN1*: Cerebellin-precursor
*CDC42EP3*: CDC42 Effector Protein 3
*CLCN4*: Chloride Voltage-Gated Channel 4
*CLIP2*: CAP-Gly Domain Containing Linker Protein 2
*COL1A2*: Collagen Type I Alpha 2 Chain
DEGs: Differentially Expressed Genes
*DENND2A*: DENN Domain Containing 2A
*DKK1*: Dickkopf WNT Signaling Pathway Inhibitor 1
*DMRT1*: Doublesex And Mab-3 Related Transcription Factor 1
*Ecm1*: Extracellular Matrix Protein 1
*EGFLAM*: EGF Like, Fibronectin Type III And Laminin G Domains
*EGFR*: Epidermal Growth Factor Receptor
ERK: Extracellular signal-Regulated Kinases
*FHL2*: Four And A Half LIM Domains 2
*FOXE1*: Forkhead Box E1
*GALNT16*: Polypeptide N-Acetylgalactosaminyltransferase 16
GDF7: Growth Differentiation Factor 7
*GPR56*: Adhesion G-Protein Coupled Receptor G1
HFG: Hand-Foot-Genital
*HOXA13*: Homeobox A1
*HTRA3*: HtrA Serine Peptidase 3
*LAPTM4B*: Lysosomal Protein Transmembrane 4 Beta
*LIM1*: LIM Homeobox 1
*LMCD1*: LIM nd Cysteine Rich Domains 1
lncRNA: Long non-coding RNA
*LOXL2*: Lysyl Oxidase Like 2
*LRIG3*: Leucine Rich Repeats And Immunoglobulin Like Domains 3
*PRUNE2*: Prune Homolog 2 With BCH Domain
MRKH: Mayer-Rokitansky-Küster-Hauser syndrome
*NEBL*: Nebulette
*OSR1*: Odd-Skipped Related Transcription Factor 1
*Pax2*: Paired Box 2
PCA: Principle Component Analysis
*PI15*: Peptidase Inhibitor 15
*POSTN*: Periostin
PPI: Protein Protein Interaction
*PRDM6*: PR/SET Domain 6
*PRICKLE1*: Prickle Planar Cell Polarity Protein 1
RA: Retinoic Acid
*RUNX1*: Runt-Related Transcription Factor 1
*SEMA*: Semaphorin
*SLIT1*: Slit Guidance Ligand 1
*SMARCA2*: SWI/SNF Related, Matrix Associated, Actin Dependent Regulator Of Chromatin, Subfamily A, Member 2
*SPOCK1*: SPARC (Osteonectin), Cwcv And Kazal Like Domains Proteoglycan 1
*SYNOP2*: Synaptopodin 2
*TGFB1*: Transforming Growth Factor Beta 1
*TSHZ3*: Tea-shirt Zinc Finger Homeobox 3
*VEGFD*: Vascular Endothelial Growth Factor D
WGCNA: Weighted Gene Co-expression Network Analysis
*WNT*: Wingless-related integration site
