# Supplementary figures and images for: Transcriptional landscape of the embryonic chicken Müllerian duct
Source: BMC Genomics. 2020 Oct 2;21:688. doi: 10.1186/s12864-020-07106-8 (PMC7532620; doi:10.1186/s12864-020-07106-8)

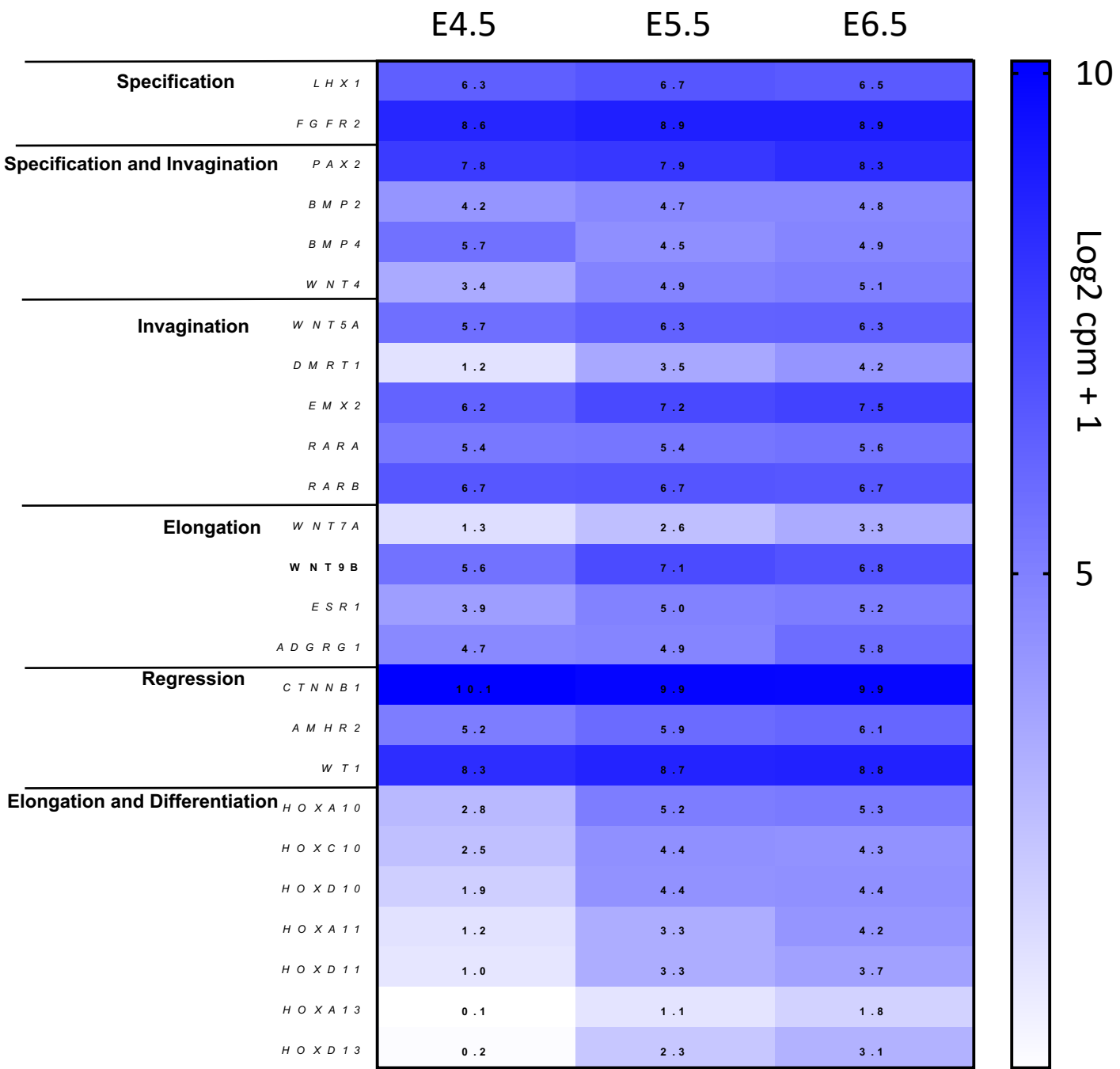

Supplement: Supplementary file 1 — Additional file 1: Supplementary Figure 1. Heatmap plot, showing expression of known genes implicated in the stages of Müllerian duct development (specification, invagination, elongation and differentiation) (Log2 cpm). Transcripts of known genes such as PAX2, LIM1 (LHX1), WNT4, DMRT1 were all present and enriched in the datasets. [file 12864_2020_7106_MOESM1_ESM.pdf]

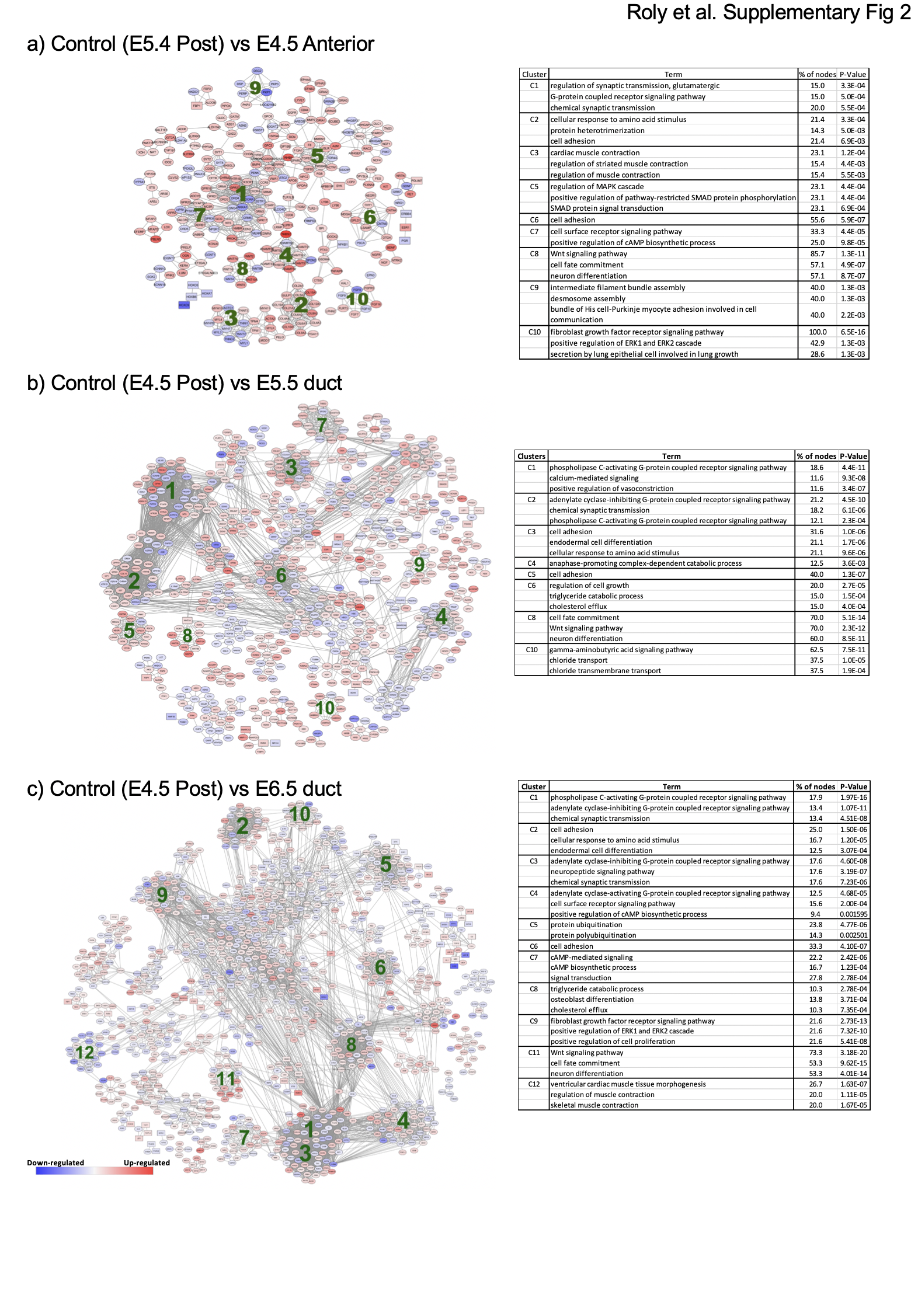

Supplement: Supplementary file 2 — Additional file 2: Supplementary Figure 2. PPI network analysis and Gene ontology of the clusters (sub-networks) for static comparison of samples. [file 12864_2020_7106_MOESM2_ESM.tif]

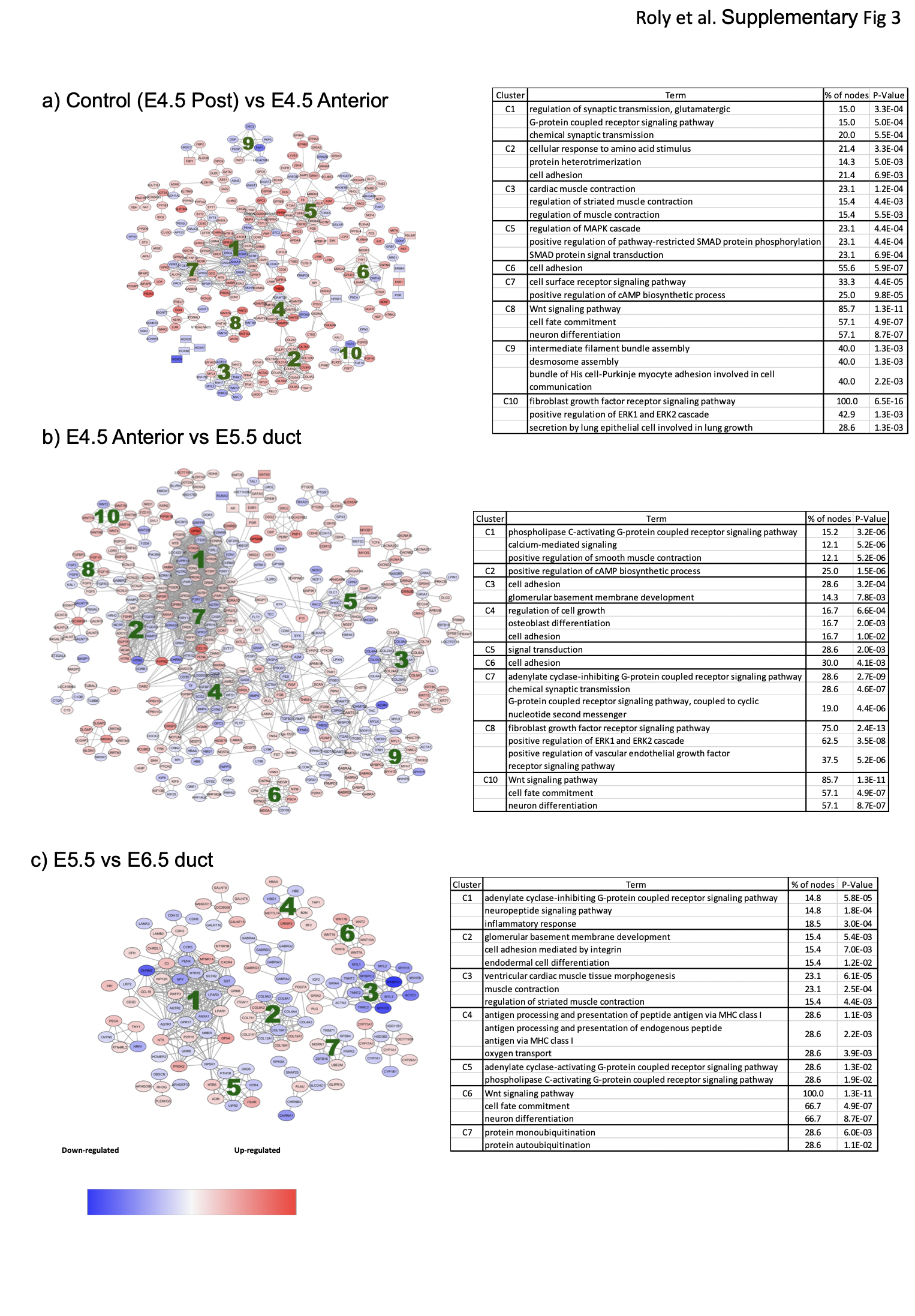

Supplement: Supplementary file 3 — Additional file 3: Supplementary Figure 3. PPI network analysis and Gene ontology of the clusters (sub-networks) for dynamic comparison of samples. [file 12864_2020_7106_MOESM3_ESM.tif]

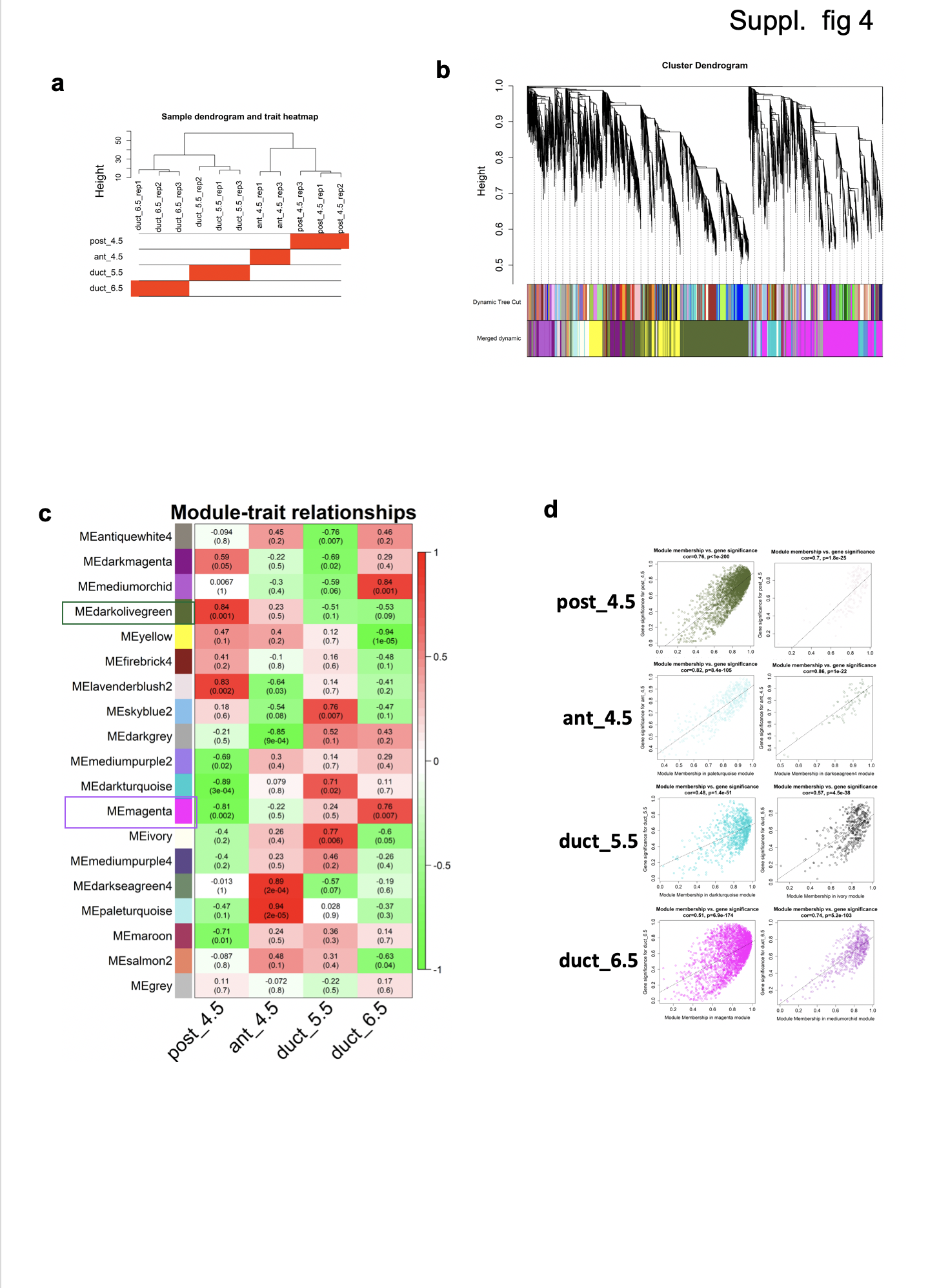

Supplement: Supplementary file 4 — Additional file 4: Supplementary Figure 4. Weighted Gene Co-expression Network Analysis. a) Hierarchical clustering of gene expression groups of individual samples by developmental stage. b) Clustering and merging modules with 75% similarity. c) Module heatmap. Expression 18 colour-coded modules (Red = high level expression, green = low level expression). Some modules positively correlated with duct development (e.g, magenta, highlighted), while others were negatively correlated (e.g., dark olive green). Post_4.5 = negative control tissue. d) Gene significance vs module membership plots of positively correlated modules with each stage. [file 12864_2020_7106_MOESM4_ESM.tif]

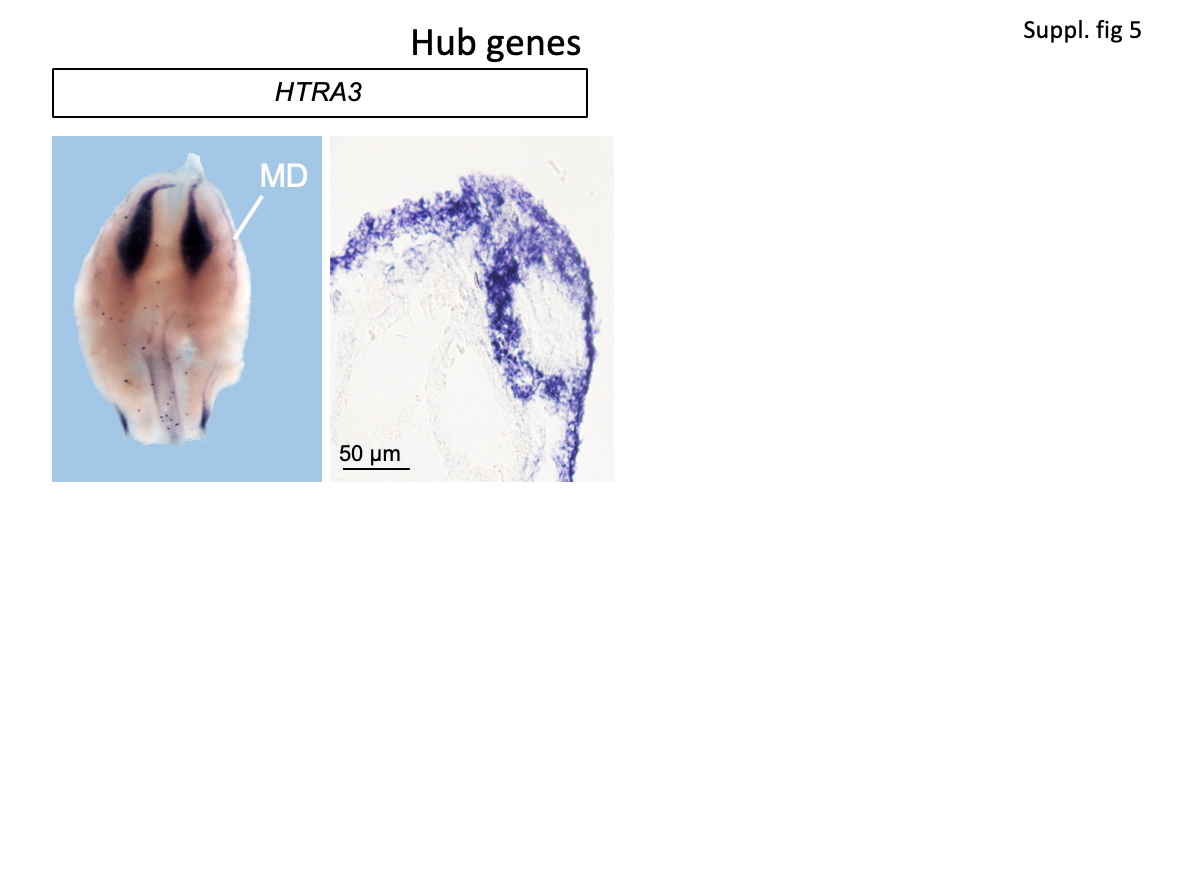

Supplement: Supplementary file 5 — Additional file 5: Supplementary Figure 5. Expression of a selected hub gene, HTRA3, in chicken Müllerian duct. The gene is expressed in the Müllerian ducts and in the anterior region of the mesonephros. Sectioned whole mount in situ’s showed expression in the mesenchymal compartment. [file 12864_2020_7106_MOESM5_ESM.tif]
