# Supplementary material for: Transcriptional landscape of the embryonic chicken Müllerian duct
Source: BMC Genomics. 2020 Oct 2;21:688. doi: 10.1186/s12864-020-07106-8 (PMC7532620; doi:10.1186/s12864-020-07106-8)
Supplement: Supplementary file 6 — Additional file 6: Supplementary Figure 6. RT-PCR analysis. Un-cropped gel images of RT-PCR analysis shown in Fig. 5b. [file 12864_2020_7106_MOESM6_ESM.pdf]

# Suppl Fig .6

## Original gel Image for fig 5.

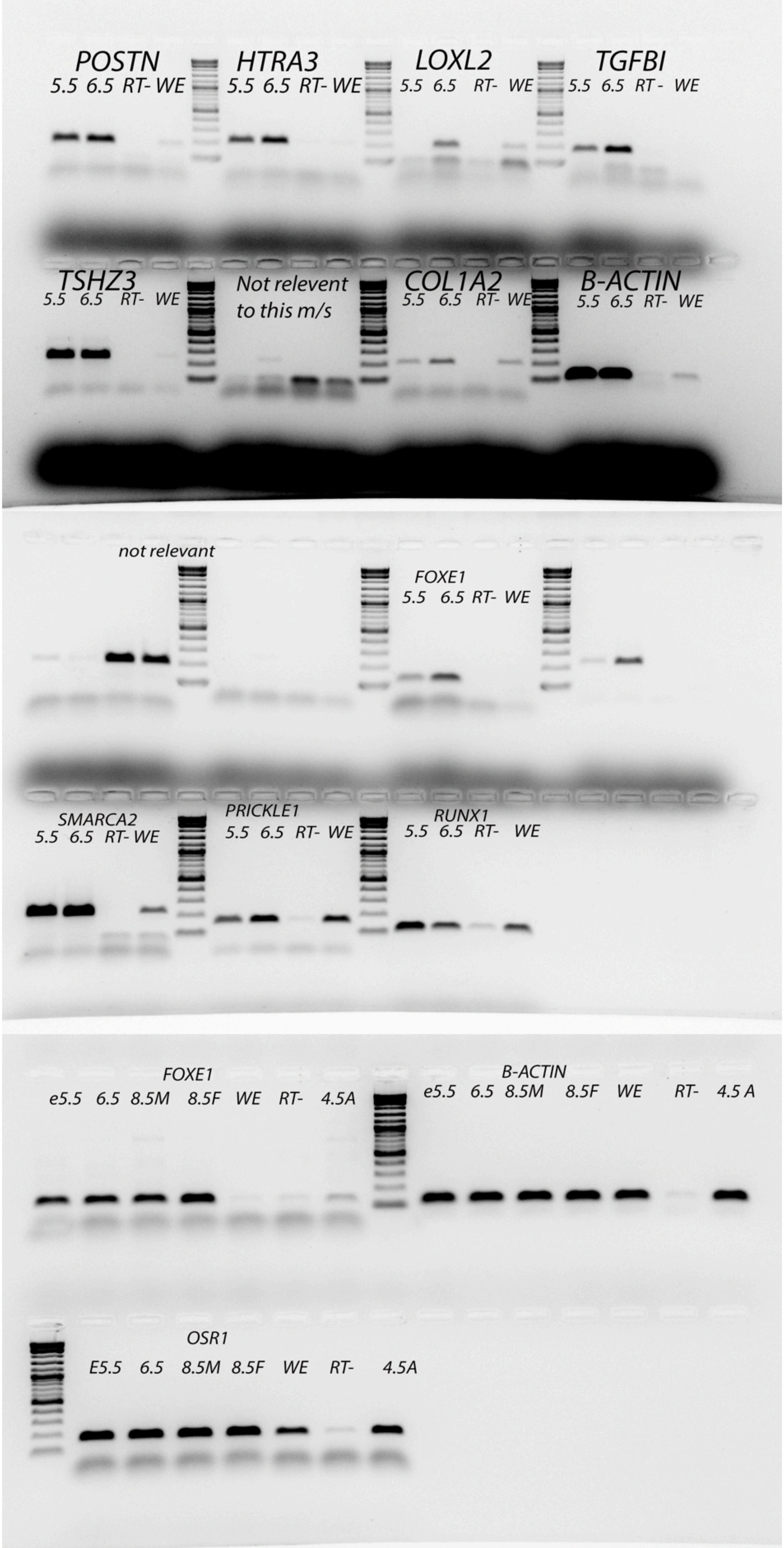


5.5 = Embryonic day 5.5 duct
6.5 = embryonic day 6.5 duct
8.5M = day 8.5 male duct
8.5F = day 8.5 female duct
RT- = Reverse Transcriptase omitted.
WE = Whole embryo, E4.5
