# Supplementary material for: Transcriptional landscape of the embryonic chicken Müllerian duct
Source: BMC Genomics. 2020 Oct 2;21:688. doi: 10.1186/s12864-020-07106-8 (PMC7532620; doi:10.1186/s12864-020-07106-8)
Supplement: Supplementary file 7 — Additional file 7. [file 12864_2020_7106_MOESM7_ESM.pdf]

G12 | PCR Primers Used in This Stud

Suppl. Table 1. PCR Primers used in this S	Forward sequence (5’→3’	Reverse sequence (5’→3’)	Application
Chicken *β-Actin*	GCTACAGCTTCACCACCACA	TCTCCTGCTCGAAATCCAGT	RT-PCR
Chicken *COL1A2*	TCAGCCTTCTCTCAGACCCA	TCCAGTAGAAACCGCTGCTC	RT-PCR
Chicken SMARCA2	GAAACGGACCCTTCCTGGTA	CACTCATATCTGGGGTTTGGC	RT-PCR
Chicken *POSTN*	GGAGCTGGAACTGAGTACACC	TTGCTGATCCCCCTTGTGTC	RT-PCR
Chicken *PRICKLE*	CGCCTCTCCGCTGTCAATG	CAGGGGCACAAGTGTGAAGT	RT-PCR
Chicken *HTRA3*	TGGCAAAGAACTTGGCTTGC	AATGCGGTCCGACGGAATAG	RT-PCR
Chicken *TGFBI*	CCACAGGGGGAACTCAACAA	TGTCTCCCTGCATGGACTTG	RT-PCR
Chicken *TSHZ3*	GGATCTCTGGGGCTAAAGCAA	TTTGCAGAAGGCTCTCCATCA	RT-PCR
Chicken RUNX1	AACCCAGAAACACGAGGCAA	CCGTCAAGTCAGAAGCACCT	RT-PCR
Chicken *FOXE1*	CGGTCAGCCAGGAGTAAGAC	GAGGACAGTCAAGTTGCCCA	RT-PCR/q-RT PCR
Chicken *LOXL2*	AGCACAACCTACCACTAACCC	CAGCACGATGAAACAGTGGT	RT-PCR
Chicken OSR1	CAGGCACTTCACCAAGTCGT	GGATATATCTGTGGTCTCTCAGGT	RT-PCR
Chicken *COL1A2*	TGGAGCAGCGGTTTCTACTG	GGCCAATGTGCAAACCGAAT	Riboprobe synthesis
Chicken *SMARCA2*	GTTTGGGAAGCGGAGGGAAT	CTGTACAGGGCTGAAAGGGG	Riboprobe synthesis
Chicken *POSTN*	TTGGAAGTGGCTTTGAGCCT	GGTGCTTCTTCCAAACGGAC	Riboprobe synthesis
Chicken *PRICKLE*	CGCCTCTCCGCTGTCAATG	AAAACTGCAACTTCACCGCC	Riboprobe synthesis
Chicken *HTRA3*	ATCGGGTCACCTACAGTCCA	TTCGGATGCCAATGAAACGC	Riboprobe synthesis
Chicken *TGFBI*	GAACTTGAACAGGCCGGGTA	CACTCTGGGAGCCTGGTTTT	Riboprobe synthesis
Chicken RUNX1	GGCAGAGGGTTGTAACCATGA	CCGTCAAGTCAGAAGCACCT	Riboprobe synthesis
Chicken *FOXE1*	TCTGCTGTTCGTGACTTTGC	GCAGATCTCTGTCCCAAACC	Riboprobe synthesis
Chicken OSR1	ATGTCATCCAGCCCAAGCAA	TCTCGGGGAGATTTCGCTCT	Riboprobe synthesis
LOC107052410	GTAATACGACTCACTATAGGGattttccagtgtt	CAATTAACCCTCACTAAAGGGtgtcctttccaacacttaatttt	Riboprobe synthesis
