# Supplementary material for: Transcriptional landscape of the embryonic chicken Müllerian duct
Source: BMC Genomics. 2020 Oct 2;21:688. doi: 10.1186/s12864-020-07106-8 (PMC7532620; doi:10.1186/s12864-020-07106-8)
Supplement: Supplementary file 8 — Additional file 8. [file 12864_2020_7106_MOESM8_ESM.pdf]

Supplementary Table 2. GO result for detected modules with WGCNA through development of Mullerian duct.

Modules	Term	Count	%	P-Value	Genes
darkolivegreen	GO:0006457~protein folding	28	1.48	0.00	HSP90AB1, GRPEL1, PDIA3, TXN2, FKBP4, PPIL2, CANX, CDC37, DNAJB11, HSPE1, NUDC, HSPA9, DNAJA2, TXNL1, P4HB, TCP1, H
	GO:0006281~DNA repair	26	1.38	0.00	BLM, RPA1, PRPF19, RPA2, FANCI, NPM1, USP10, ERCC3, ERCC4, ASF1A, FANCC, EXO1, SSRP1, NUDT1, USP1, PIF1, DDB1, ATR, XF
	GO:0006260~DNA replication	23	1.22	0.00	ING5, SSRP1, SSBP1, BLM, TICRR, DTL, PIF1, LIG3, RBBP7, RMI1, RPA3, RPA1, TOP1, DNA2, RPA2, RFC3, RRM1, CHAF1A, CHAF1B,
	GO:0030433~ER-associated ubiquitin-dependent protein catabolic process	17	0.90	0.00	DERL2, MAN1B1, CCDC47, ERLIN2, EDEM3, UBE2J2, EDEM1, STT3B, HSP90B1, PSMC6, SEC61B, PSMC5, PSMC3, PSMC2, PSMC1,
	GO:0006606~protein import into nucleus	15	0.79	0.00	NUP133, NUP153, CSE1L, NUP88, RAN, SNUPN, NUP50, NUP93, RANBP1, NUP107, KPNA4, NUP155, KPNA2, TNPO3, NUP58
	GO:0000462~maturation of SSU-rRNA from tricistronic rRNA transcript	14	0.74	0.00	KRR1, TBL3, TSR1, UTP18, BYSL, MRPS11, UTP6, HEATR1, DIEXF, FCF1, DCAF13, MRPS9, WDR3, UTP20
	GO:0007059~chromosome segregation	14	0.74	0.00	KIF11, FAM96A, NEK2, NDC80, RCC1, KNSTRN, SRPK1, BRCA1, MIS12, SPC25, TOP1, INCENP, SKA2, TOP2A
	GO:0032543~mitochondrial translation	11	0.58	0.00	MRPL2, QRSL1, MRPL23, MRPS18A, PTCD3, CHCHD1, HARS, MTERF3, MRPL47, MRPS7, GATC
	GO:0000387~spliceosomal snRNP assembly	11	0.58	0.00	CLNS1A, STRAP, SNRPD3, SNRPD1, LSM4, SNRPC, DDX20, GEMIN6, SNRPE, SNRPG, GEMIN5
	GO:0006270~DNA replication initiation	10	0.53	0.00	CDC7, CCNE2, PPP1R3E, ORC4, POLA1, ORC6, MCM10, MCM4, MCM5, MCM6
	GO:0042273~ribosomal large subunit biogenesis	10	0.53	0.00	ZNF622, EIF6, EBNA1BP2, SDAD1, SURF6, NOP16, NIP7, NLE1, AAMP, NOC2L
	GO:0006099~tricarboxylic acid cycle	10	0.53	0.00	SDHA, DLST, ACO2, SUCLG1, OGDHL, MDH2, PDHB, IDH3A, FH, MDH1
lavenderblush2	GO:0035556~intracellular signal transduction	9	5.63	0.00	DEPDC6, PRKCZ, PDZD8, SGK1, NRG4, PSEN2, ASB1, OXSR1, PRKAA2
	GO:0090303~positive regulation of wound healing	3	1.88	0.00	ANXA1, ARFGEF1, KANK1
	GO:0060993~kidney morphogenesis	3	1.88	0.01	LHX1, GDNF, GCNT1
paleturquoise	GO:0007155~cell adhesion	11	2.74	0.01	NCAM2, ITGA9, CD9, VWF, TNC, EFNB2, ITGA1, NINJ1, RHOB, THBS2, PARVB
	GO:0007229~integrin-mediated signaling pathway	6	1.49	0.01	ITGA9, PTK2, PLEK, FYN, ITGB8, ITGA1
darkseagreen4	GO:0007015~actin filament organization	3	4.17	0.01	TMSB15B, LMOD1, TPM2
	GO:0003161~cardiac conduction system development	2	2.78	0.01	CDC42, GJA5
darkturquoise	GO:0035556~intracellular signal transduction	21	2.55	0.01	BRSK2, SOCS6, DSTYK, DAPK2, DEPDC5, HUNK, PLCL1, TULP4, PLCB4, MAST2, NOD1, AKAP6, DCLK2, ASB3, JAK2, PLCD1, GUCY1
	GO:0007224~smoothened signaling pathway	8	0.97	0.01	KIF3A, TTC21B, CC2D2A, HHAT, ROR2, IFT27, BOC, PTPDC1
	GO:0032007~negative regulation of TOR signaling	6	0.73	0.00	HIF1A, TSC1, SIRT1, NPRL3, FNIP1, DEPDC5
	GO:0048666~neuron development	6	0.73	0.01	TENM1, SECISBP2, SKI, PBX3, THOC2, TGFB2
	GO:0061512~protein localization to cilium	5	0.61	0.00	TULP4, CSNK1D, TTC21B, TBC1D32, SNX10
ivory	GO:0045944~positive regulation of transcription from RNA polymerase II promoter	25	6.203474	0.00	CCNT2, ARID4A, CREM, ARID4B, BMPR2, PRKDC, RORA, GLI3, NR2C2, PHIP, AGO1, PPP1R12A, PKD1, BCL9L, ZBTB49, KLF12, RBM
	GO:0060021~palate development	7	1.736973	0.00	ACVR2B, MEOX2, TGFBR1, LRP6, WNT11, GLI3, BMPR1A
	GO:0009952~anterior/posterior pattern specification	7	1.736973	0.00	ACVR2A, ACVR2B, TSHZ1, BPTF, TGFBR1, BMPR2, GLI3
	GO:0001756~somitogenesis	6	1.488834	0.00	EP300, PSEN1, PLXNA2, PRKDC, ATM, BMPR1A
	GO:0045669~positive regulation of osteoblast differentiation	6	1.488834	0.00	PRKD1, ACVR2A, ACVR2B, BMPR2, GLI3, BMPR1A
	GO:0007519~skeletal muscle tissue development	5	1.240695	0.00	CCNT2, EP300, MEOX2, SVIL, FOXP1
	GO:0048041~focal adhesion assembly	4	0.992556	0.00	PTPRK, ARHGAP6, ARHGEF7, ACTN2
	GO:0034454~microtubule anchoring at centrosome	3	0.744417	0.00	NIN, PCM1, HOOK3
	GO:0060840~artery development	3	0.744417	0.01	ACVR2B, BMPR2, GLI3
	GO:0034773~histone H4-K20 trimethylation	3	0.744417	0.01	ARID4A, ARID4B, KMT5B
	GO:0050768~negative regulation of neurogenesis	3	0.744417	0.01	PCM1, BMPR1A, HOOK3
magenta	GO:0000122~negative regulation of transcription from RNA polymerase II promoter	46	2.75	0.00	BMI1, FGFR1, FGF9, FHL2, NFIX, CBX8, SUFU, HIC1, KDM1A, TSC22D3, PCGF2, ATN1, OSR1, MYOCD, NRARP, JUND, BCL6, NR2F2,
	GO:0007165~signal transduction	35	2.09	0.01	TLR5, APBB1IP, SUFU, LINGO1, SMOC2, CRY2, ANK1, MYD88, UNC5B, NDRG4, UNC5A, MIER1, FGB, PDE1A, PDE4B, TICAM1, UNC
	GO:0010628~positive regulation of gene expression	24	1.43	0.00	LRRC32, FGF9, PIK3CD, SLC6A4, MST1, ESR1, PRKAB1, RNF207, TLE1, MAPK11, HGF, KIT, SHH, GLI1, NTRK3, ACVR1B, SPRY2, ID2,
	GO:0030335~positive regulation of cell migration	20	1.19	0.00	WNT5B, NTF3, PDGFB, PDGFA, PIK3CD, SUN2, LEF1, HGF, NTRK3, DAB2, SEMA6D, SEMA3F, SEMA7A, NUMB, SEMA3D, CEMIP, PD
	GO:0005975~carbohydrate metabolic process	16	0.96	0.01	HYAL1, B4GALT2, GLB1L, HEXA, CHST3, HEXDC, B3GAT1, PGM5, HPSE2, CHST6, GALC, GAA, HHIP, GPD1L2, AMY1A, PYGB
	GO:0090090~negative regulation of canonical Wnt signaling pathway	15	0.90	0.01	EGR1, WNT5A, NKD1, DAB2IP, WNT5B, DRAXIN, LEF1, CDH2, ISL1, SHH, DKK3, NPHP4, DAB2, PRICKLE1, MAD2L2
	GO:0001755~neural crest cell migration	13	0.78	0.00	ANXA6, SMO, SEMA6D, EFNB1, SEMA3F, SEMA7A, SEMA3D, LEF1, SEMA3A, HTR2B, ISL1, SHH, ALX1
	GO:0043433~negative regulation of sequence-specific DNA binding transcription factor activity	10	0.60	0.00	CTNNBIP1, HDAC4, KDM1A, PDLIM7, ID2, PIM1, ESR1, NR0B1, MAD2L2, SUFU
	GO:0060348~bone development	9	0.54	0.00	SMAD9, IFT172, ASXL1, ANKRD11, NPR2, PDGFC, SMAD1, SPARC, PAPSS2
	GO:0042472~inner ear morphogenesis	9	0.54	0.01	FGFR1, SPRY2, ABR, FGF9, ALDH1A3, ITGA8, PRRX1, SOBP, NTN1
	GO:0032091~negative regulation of protein binding	9	0.54	0.01	CTNNBIP1, KDM1A, DAB2, PDGFB, CSNK1E, SORL1, CAMK1, ZFPM1, DVL1
	GO:0001558~regulation of cell growth	9	0.54	0.01	CLSTN3, KAZALD1, NANOS1, BAP1, HTRA3, IGFBP2, MAD2L2, IGFBP4, CRIM1
mediumorchid	GO:0006412~translation	14	2.64	0.00	MRPL51, RPL15, RPL27, RPS6, RPS26, SLC25A14, RPS28, RPS17, RPS3A, RPS12, SLC25A28, RPS13, RPS11, RPS20
	GO:0032880~regulation of protein localization	5	0.94	0.01	RACK1, TMEM231, SIX1, WASL, PRNP
